# Inferring Branch-Specific Rates of Lineage Diversification Under the Birth–Death-Shift Process

**DOI:** 10.1093/sysbio/syag003

**Published:** 2026-01-24

**Authors:** Sebastian Höhna, William A Freyman, Zachary Nolen, John P Huelsenbeck, Michael R May, Bruce Rannala, Brian R Moore

**Affiliations:** Department of Earth and Environmental Sciences, Paleontology and Geobiology, Ludwig-Maximilians-Universität München, Richard-Wagner-Str. 10, 80333 Munich, Germany; GeoBio-Center LMU, Ludwig-Maximilians-Universität München, Richard-Wagner-Str. 10, 80333 Munich, Germany; Department of Ecology, Evolution, and Behavior, University of Minnesota, Twin Cities, 1479 Gortner Avenue, Saint Paul, MN 55108, USA; Department of Biology, Lund University, Sölvegatan 37, 223 62 Lund, Sweden; Department of Integrative Biology, University of California, Berkeley, 3060 VLSB #3140, Berkeley, CA 94720-3140, USA; Department of Evolution and Ecology, University of California, Davis, Storer Hall, One Shields Avenue, Davis, CA 95616, USA; Department of Evolution and Ecology, University of California, Davis, Storer Hall, One Shields Avenue, Davis, CA 95616, USA; Department of Evolution and Ecology, University of California, Davis, Storer Hall, One Shields Avenue, Davis, CA 95616, USA

**Keywords:** Birth–death process, lineage-diversification rates, phylogeny, RevBayes

## Abstract

Inferring how rates of speciation and extinction vary across lineages has proven to be a difficult statistical problem. Here, we describe a stochastic-diversification model—called the birth–death-shift (BDS) process—in which diversification rates may vary across both extant and extinct and unsampled lineages. We estimate the parameters of this model in a Bayesian statistical framework from phylogenies of exclusively extant lineages. We perform simulation studies to validate the implementation of our method and to characterize its statistical behavior. We also perform analyses of an empirical primates data set, which reveal that estimates of branch-specific diversification rates are robust to the assumed prior distribution on the number of diversification-rate shifts. Our implementation of the BDS model in RevBayes provides biologists with a flexible approach for estimating branch-specific diversification rates under a statistically coherent model.

Variable rates of lineage diversification—speciation and extinction—are central to many fundamental evolutionary processes. Diversification rates may vary in three distinct ways. First, rates may vary in a *time-dependent* manner where every lineage has an identical diversification rate at any given instant, but those rates vary over time. For example, mass-extinction events involve a simultaneous, tree-wide spike in extinction rate across all lineages (Raup 1979; Raup and Sepkoski 1982; Jablonski 2008; Culshaw et al. 2019). Second, rates may vary in a *state-dependent* manner, where the diversification rate of each lineage depends on the state of some variable, such as the state of a morphological trait (Paradis 2005; Maddison et al. 2007). For example, key innovations increase speciation rates and/or decrease extinction rates of lineages in which they arise (Hunter 1998), such as herbivory in beetles (Mitter et al. 1988) or the nectar spurs of columbines (Hodges and Arnold 1995). Finally, rates may vary in a *lineage-specific* manner where diversification rates vary across lineages. For example, adaptive radiations involve groups that diversify at exceptionally high rates (Seehausen 2006; Gavrilets and Losos 2009).

Phylogenies provide an explicit history of the process of lineage diversification, and therefore provide information required to identify diversification-rate variation (Nee et al. 1994; Ricklefs 2007; Morlon 2014; Morlon et al. 2024). Accordingly, phylogenetic methods have been developed to study diversification-rate variation. For example, phylogenetic methods are available for inferring time-dependent rate variation (e.g., Stadler 2011; Höhna et al. 2016; Magee et al. 2020), including models where diversification rates are correlated with environmental changes (e.g., Condamine et al. 2018; Meseguer and Condamine 2020; Palazzesi et al. 2022) or episodes of mass extinction (e.g., Höhna 2015; May et al. 2016; Culshaw et al. 2019). Similarly, phylogenetic methods are available for inferring state-dependent variation, where diversification rates are correlated with the evolution of discrete traits (e.g., Maddison et al. 2007; FitzJohn 2012; Magnuson-Ford and Otto 2012; Beaulieu and O’Meara 2016; Freyman and Höhna 2018), with the evolution of continuous traits (FitzJohn 2010), or with changes in species range (e.g., Goldberg et al. 2011; Landis et al. 2022). Finally, phylogenetic methods are available for inferring lineage-specific rate variation (e.g., Chan and Moore 2002, 2005; Alfaro et al. 2009; Morlon et al. 2011; Jetz et al. 2012; Rabosky 2014; Maliet et al. 2019; Barido-Sottani et al. 2020; Maliet and Morlon 2022; Vasconcelos et al. 2022; Barido-Sottani and Morlon 2023; Mazet et al. 2023; Quintero et al. 2024; Kopperud and Höhna 2025).

The general outline of phylogenetic methods for inferring diversification-rate variation is as follows (see also Morlon et al. 2024). We first develop a probabilistic model capable of generating phylogenies under a process where diversification rates vary in the relevant manner (i.e., with parameters that allow diversification rates to vary in a time-dependent, state-dependent, or lineage-specific manner). A (diversification) model is statistically coherent if it faithfully corresponds to the process used to generate data, that is, the assumptions match the generative process and the probability distribution of the data is correctly calculated. This is a critical property, as it allows us to calculate the probability of observing a study tree under a specified diversification model. Correctly calculating the probability of a study tree, in turn, provides the foundation for likelihood-based (ML or Bayesian) inference of the model parameters. In maximum-likelihood estimation, for example, we find the set of parameter estimates that maximizes the probability of observing the data (our study tree) under the diversification model. By contrast, in a Bayesian framework, we estimate the joint posterior probability distribution of the model parameters (reflecting our beliefs in the model parameters after evaluating our study data), which is achieved by averaging the likelihood over the joint prior probability distribution for our model parameters.

Lineage diversification models are a type of Markov process, where the probability of the next state of the process depends only on the current state of the process. For example, in the constant-rate birth–death process (Nee et al. 1994), the process’s state changes according to two types of events: speciation events that occur at rate $\lambda$ (the speciation rate) where a single lineage gives rise to two daughter lineages, and extinction events that occur at rate $\mu$ (the extinction rate) where a single lineage is terminated. Lineage-specific birth–death-shift (BDS) processes must additionally model how the speciation and/or extinction rates vary among lineages; for example, lineages may experience diversification-rate shifts that induce a random change in the speciation and/or extinction rates according to a base distribution. It is important to note that diversification-rate shifts are modeled jointly with the processes that generate the tree because—unlike most characters that evolve passively over the branches of a tree—diversification-rate shifts directly impact the growing phylogeny. Note also that under any birth–death process, including a lineage-specific BDS process, a lineage that is extant at some point in the past may go extinct before the present. Indeed, diversification-rate shifts will impact the probability of whether a lineage at some point in the past leaves extant descendants. Similarly, a lineage at some point in the past may or may not have descendants that are included in the sample of study lineages. Accordingly, diversification-rate shifts occurring on lineages that go extinct before the present and/or are not sampled in the study phylogeny are an intrinsic property of this general process. Computing the likelihood of a study tree under a lineage-specific diversification model therefore requires accounting for diversification-rate shifts on both extant and extinct/unsampled branches (Moore et al. 2016; Barido-Sottani et al. 2020), which is a significant mathematical and/or computational challenge (Maliet and Morlon 2022).

Several methods have been proposed to infer lineage-specific diversification-rate variation (e.g., Alfaro et al. 2009; Morlon et al. 2011; Jetz et al. 2012; Rabosky 2014; Maliet et al. 2019; Barido-Sottani et al. 2020; Maliet and Morlon 2022; Vasconcelos et al. 2022; Barido-Sottani and Morlon 2023; Mazet et al. 2023; Quintero et al. 2024; Kopperud and Höhna 2025). Despite considerable recent progress on this problem, we believe that there are opportunities for improvement. To this end, we present a novel Bayesian approach for inferring lineage-specific rates of speciation and extinction from phylogenies of extant taxa. The novelty of our approach is that it uniquely combines several beneficial attributes: it is based on a statistically coherent lineage-specific diversification process; it conforms to our biological understanding of how diversification rates evolve across lineages; and it is implemented in a manner that correctly calculates the probability of observed trees, is computationally efficient, enables specification of a large family of lineage-specific diversification models, and allows us to objectively assess the fit of alternative diversification models to a given study tree. More specifically, our approach is based on a novel BDS process that models lineage-specific diversification-rate variation as shifts among a set of discrete speciation- and extinction-rate categories. Our approach involves two main components: 1) we calculate likelihoods under our BDS process by adapting previous theoretical work on inferring state-dependent diversification-rate variation (Maddison et al. 2007; FitzJohn 2012; Caetano et al. 2018); and 2) we map diversification histories over the branches of the phylogeny by adapting previous theoretical work on stochastic mapping (Nielsen 2002; Freyman and Höhna 2019). Our approach distinguishes itself from these existing approaches in several key aspects. i) Our approach aims to infer branch-specific diversification rates over the entire phylogeny instead of only estimating tip rates (Jetz et al. 2012; Vasconcelos et al. 2022). ii) Our approach models diversification-rate shifts as events occurring along a lineage (as done by Rabosky 2014; Barido-Sottani et al. 2020) instead of at speciation events (Maliet et al. 2019; Maliet and Morlon 2022; Barido-Sottani and Morlon 2023) or ad hoc without an underlying process (Alfaro et al. 2009; Morlon et al. 2011; Mazet et al. 2023). iii) Our approach draws diversification rates at diversification-rate shift events from a finite set of rates (as done by Barido-Sottani et al. 2020; Kopperud and Höhna 2025) instead of fully continuous base distributions (Rabosky 2014; Maliet et al. 2019; Maliet and Morlon 2022; Barido-Sottani and Morlon 2023; Quintero et al. 2024) or ad hoc without an underlying process (Alfaro et al. 2009; Morlon et al. 2011; Mazet et al. 2023). iv) Most importantly, our approach accounts for diversification-rate shifts at extinct or unsampled lineages (as done by Maliet et al. 2019; Quintero et al. 2024; Kopperud and Höhna 2025) instead of assuming some prescient process that implicitly conditions on no diversification-rate shifts at extinct or unsampled lineages (Alfaro et al. 2009; Morlon et al. 2011; Rabosky 2014; Mazet et al. 2023). Finally, our approach is most similar to the multitype birth–death (MTBD) process by Barido-Sottani et al. (2020), except that MTBD by default does not account for diversification-rate shifts at extinct and unsampled lineages by virtue of its greater computational efficiency.

We validate the implementation of our method and characterize its statistical behavior by means of a simulation study. Finally, we demonstrate our method with an empirical analysis of primates. All of the methods described in this paper have been implemented in the Bayesian phylogenetic inference software package RevBayes (Höhna et al. 2016).

## Bayesian Inference of Branch-Specific Diversification Rates

### Overview

Our goal is to develop a Bayesian approach for inferring the diversification rate of every branch in a study tree. Central to any such method is the ability to correctly compute the probability of observing the phylogenetic data under this model (i.e., the likelihood); this probability allows us to estimate the parameters of interest. Here, the data correspond to a time-calibrated phylogeny of the study group consisting solely of extant lineages, and the focal quantity is the diversification rate of every branch. Conceptually, calculating the probability of a study tree under the BDS process requires either data augmentation of the diversification-rate history and all extinct and unsampled lineages (Maliet and Morlon 2022) or computing the probability by integrating over all possible diversification-rate histories and extinct and unsampled lineages. The integration would be impossible for continuous speciation and extinction rates because every calculation would entail integrating over an infinite number of continuous parameter values where analytical solutions for such an integral are not known. We therefore consider a finite number of discrete diversification-rate categories (see below).

Our approach has two components: a postorder (tip-to-root) algorithm that calculates the likelihood of the study tree under the BDS process and a preorder (root-to-tip) algorithm that maps diversification histories over the branches of the study tree. The first component of our method draws heavily on the numerical methods developed for calculating likelihoods under state-dependent speciation and extinction (SSE) models (Maddison et al. 2007; FitzJohn 2012). Instead of calculating likelihoods under a process where the diversification rates depend on the observed states of a discrete character, our approach involves calculating likelihoods under a process where diversification rates depend on a hidden state corresponding to a particular rate category. This numerical method calculates the likelihood of our observed study tree under the BDS process by averaging over all possible histories of lineage-specific diversification rates. In so doing, however, this procedure also integrates out the focal quantity: the speciation and extinction rate of each branch in the tree.

Accordingly, the second component of our method leverages stochastic-mapping algorithms (Nielsen 2002; Freyman and Höhna 2019). Rather than mapping character histories where the states of a discrete trait are painted across branches of the tree, our approach involves mapping diversification histories where the discrete speciation- and extinction-rate categories are painted across branches of the tree. Our method is implemented in a Bayesian statistical framework, so we use a Markov chain Monte Carlo (MCMC) algorithm that samples parameter values with a frequency that is proportional to their posterior probability.

### The BDS Process

Our BDS process is based on the familiar constant-rate birth–death process (Kendall 1948; Thompson 1975; Yule 1925). The constant-rate birth–death process involves two types of events: speciation events, where a single lineage gives rise to two daughter lineages, and extinction events, where a single lineage is terminated. In a small interval of time, $\Delta t$, a lineage will experience a speciation event with probability $\lambda \Delta t$, or go extinct with probability $\mu \Delta t$. As a Markov process, the waiting times between these events are exponentially distributed, where the rate parameter is $\lambda + \mu$. When an event occurs, it will be a speciation event with probability $\lambda / (\lambda +\mu )$ or an extinction event with probability $\mu / (\lambda + \mu )$.

We extend the constant-rate birth–death process by adding a third type of event: diversification-rate-shift events, where a single lineage experiences a change in the speciation and/or extinction rates. During a rate-shift event, the affected lineage draws its new speciation and extinction rates from corresponding probability distributions. Biologically, we expect that rates of speciation and extinction are continuous. Nevertheless, the probability calculations for the BDS process would be impossible if we had to integrate over continuous distributions for the diversification-rate parameters. Accordingly, we adopt an approach that provides an approximation of these integrals. Under this approach, we first divide the continuous probability distributions for the diversification-rate parameters into a finite number of *K* bins. The width of each bin is defined such that each category contains equal probability, that is, we use the *K* quantiles of the underlying continuous probability distribution. The diversification rate for *i*th discrete category is the median value of the corresponding quantile. As detailed in the following section, our probability calculations involve summing over these *K* discrete diversification-rate categories.

**Figure 1.**
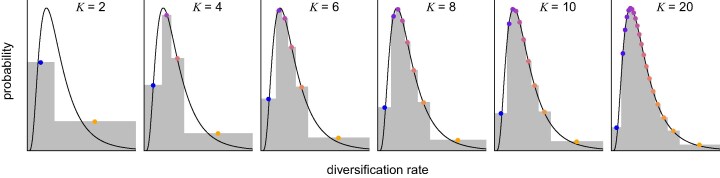
Approximating the continuous probability distributions for the diversification-rate parameters. For computational tractability, our approach for computing the probability of a tree under the BDS process involves discretizing the continuous base distributions for the speciation and extinction rates into a finite number of categories, *K*. Specifically, the continuous base distribution is divided into *K* quantiles, such that each discrete category defines an area with equal probability (gray rectangles), and the median of each quantile is the diversification rate for that category (colored dots). We compute probabilities by numerically marginalizing over the *K* discrete rate categories. This approach provides a computationally tractable alternative to computing the continuous integral, and will provide a reliable approximation of the continuous integral when the number of categories *K* is sufficiently large to approximate the underlying continuous distribution.

As in the discrete-gamma model for accommodating among-site variation in substitution rates (Yang 1994), the number of rate categories, *K*, is not a parameter of our model; that is, it is an assumption of the analysis rather than a value estimated from the data. The number of rate categories therefore represents a compromise: the approximation of the underlying continuous probability distribution improves as we increase the number of discrete categories (Fig. 1). However, the computational burden also scales with the number of discrete categories. Thus, the value of *K* is only of interest to the extent that it is sufficiently large to avoid discretization bias while remaining small enough to allow efficient computation. We will explore the impact of using different numbers of diversification-rate categories, and also demonstrate a protocol for selecting *K* in a later section.

Note that, in principle, the base distributions for the diversification-rate parameters could take many forms, provided the resulting speciation and extinction rates are non-negative (e.g., see Supplementary Material Section S2). Each unique base distribution corresponds to a different diversification model, such that there is a large family of BDS models. To simplify the presentation of our approach, we adopt here a specific base distribution. Specifically, we assume that new speciation and extinction rates are drawn independently from separate lognormal distributions with means $\theta _\lambda$ and $\theta _\mu$ and standard deviations $\sigma _\lambda$ and $\sigma _\mu$, respectively. Speciation and extinction rates are drawn independently from *K* equiprobable categories; accordingly, there are a total of ${K \times K = M}$ combinations of speciation and extinction rates, each with probability ${1 / M}$. We index a specific pair of speciation and extinction rates with ${i \in 1{:}M}$, where the speciation and extinction rates for the *i*th rate category are $\lambda _i$ and $\mu _i$, respectively.

### Calculating Likelihoods under the BDS Process

Maddison et al. (2007) introduced a generic algorithm for calculating the probability of an inferred tree under a state-dependent birth–death process. Specifically, this is a postorder (tip-to-root) algorithm that visits every point along the tree, and at each point we keep track of two probability terms: 1) the extinction probability, $E_i(t)$, which specifies the probability that a lineage in rate category *i* at time *t* goes extinct by (or is unsampled at) the present, and 2) the descendant-lineage probability, $D_{N,i}(t)$, which specifies the probability that a lineage *N* at time *t* gives rise to its observed descendant subtree, given that it begins in rate category *i* at time *t*. This algorithm involves recursively solving a set of differential equations as we move over the branches of a tree, visiting each point on the tree from its tips to the root in small time steps, $\Delta t$. The differential equations describe how these probabilities change as we move backward in time. If we start with appropriately chosen initial values for $E_i(t)$ and $D_{N,i}(t)$ at the tips of the tree, upon reaching the root of the tree these differential equations will have computed the probability of observing the study tree under the BDS process, that is, calculated the likelihood of the observed tree.

We begin at the present, ${t = 0}$, where we specify initial values for ${E_i(0)}$ and ${D_{N,i}(0)}$ for each tip. For a tree with a lineage-sampling fraction $\rho$, we initialize the probability of observing each sampled lineage as ${D_{N,i}(0)=\rho }$, and the probability of each lineage being extinct or unsampled as ${E_i(0) = 1-\rho }$ (FitzJohn et al. 2009). Note that we use the same initial values for ${E_i(0)}$ and ${D_{N,i}(0)}$ for all *M* diversification-rate categories; this is because the rate category of a lineage at the present is not observed.

**Figure 2.**
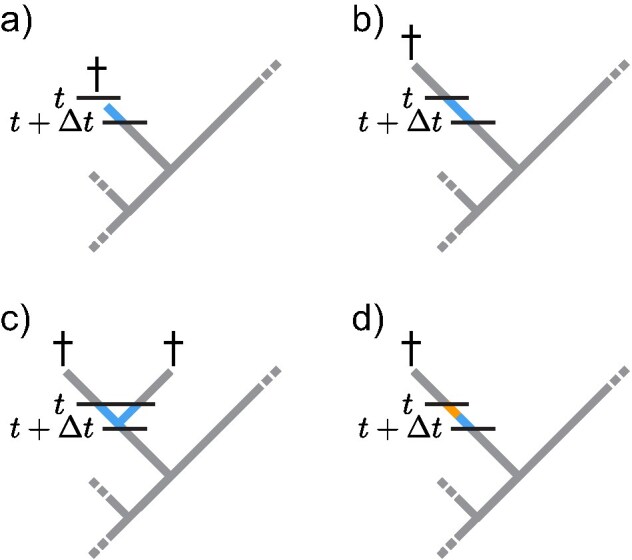
Scenarios occurring in the interval $\Delta t$ that could lead to the extinction of a lineage before the present. For each step into the past from time *t* to ($t +\Delta t$), we compute the change in the extinction probability, $E_i(t)$, by enumerating all scenarios that could occur in the interval $\Delta t$: a) the lineage goes extinct in the interval $\Delta t$; in the remaining three scenarios, the lineage does not go extinct in the interval, and b) nothing happens (no extinction, speciation, or rate shift in the interval $\Delta t$), with subsequent extinction before the present, c) the lineage speciates in the interval $\Delta t$, with subsequent extinction of both daughter lineages before the present, or d) the lineage shifts from rate category *i* to *j*, with subsequent extinction before the present.

Next, we begin our traversal of the tree from each tip (where $t=0$) to the root in small time steps, $\Delta t$. We calculate the change in the extinction probability, $E_i(t)$, for each step into the past from *t* to $(t + \Delta t)$ by enumerating all of the scenarios that could occur within the interval $\Delta t$. The differential equations describing the change in the extinction probability can be derived from finite-difference equations that enumerate the probabilities of individual events that could occur in a small time interval $\Delta t$,


(1) \begin{eqnarray*}
E_i(t{+}\Delta t) &= & \mu _i \Delta t \quad \mathrm{(A)} \\
+\, (1 - \lambda _i \Delta t - \mu _i \Delta t - \eta \Delta t) \times E_i(t) \quad \mathrm{(B)} \\
+\, \lambda _i \Delta t \times E_i(t)^2 \quad \mathrm{(C)} \\
+\, \sum _{j \ne i}^M \left(\frac{\eta \Delta t}{M-1} E_j(t)\right),\quad \mathrm{(D)}
\end{eqnarray*}

where the last term involves a sum over all other $(M-1)$ discrete rate categories. Note that it would be impossible to evaluate this sum for continuous diversification rates (it would involve summing an infinite number of terms), which is the motivation for discretizing diversification rates in our approach (Fig. 1). We also note that in a small interval of time $\Delta t$, only one event can occur under our model. The events associated with each term in this equation are described in the caption of Figure 2.

Similarly, we calculate the change in the descendant-lineage probability, $D_{N,i}(t)$, over the interval $(t + \Delta t)$ by enumerating all of the events that could occur within the interval $\Delta t$. The finite-difference equation is


(2) \begin{eqnarray*}
D_{N,i}(t{+}\Delta t) &= & (1 - \lambda _i \Delta t - \mu _i \Delta t - \eta \Delta t) \times D_{N,i}(t) \quad \mathrm{(A)} \\
+\, \lambda _i\Delta t \times D_{N,i}(t) \times E_i(t) \quad \mathrm{(B)} \\
+\, \lambda _i\Delta t \times D_{N,i}(t) \times E_i(t) \quad \mathrm{(C)} \\
+\, \sum _{j \ne i}^{M} \left(\frac{\eta \Delta t}{M-1} D_{N,j}(t)\right). \quad \mathrm{(D)}
\end{eqnarray*}

**Figure 3.**
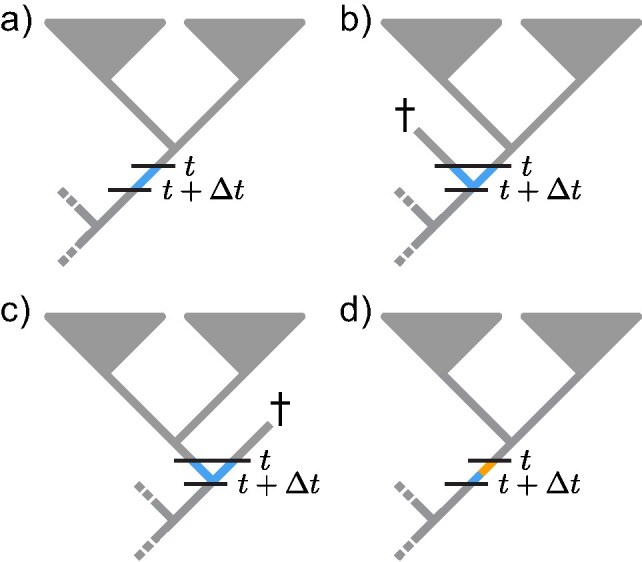
Scenarios occurring in the interval $\Delta t$ that could lead to the observed subtree. For each step into the past from time *t* to ($t +\Delta t$) we compute the change in the descendant-lineage probability, $D_{N,i}(t)$, by enumerating all of the possible scenarios that could occur in the interval $\Delta t$: a) nothing happens, and the lineage eventually becomes the observed subtree; b) the lineage speciates in the interval, the left lineage goes extinct, and the right lineage becomes the observed subtree; c) the lineage speciates in the interval, the right subtree goes extinct, and the left lineage becomes the observed subtree; or d) the lineage shifts from rate category *i* to *j*, and subsequently becomes the observed subtree.

Here, we compute the probability of no extinction occurring in the time interval, $\Delta t$, and consider the other events that can occur while still giving rise to the observed descendant subtree (described in the caption of Fig. 3).

We derive a differential equation for $D_{N,i}(t)$ from the finite-difference equation, $D_{N,i}(t+\Delta t)$, by subtracting $D_{N,i}(t)$, dividing by $\Delta t$, and taking the limit as $\Delta t \rightarrow 0$:


(3) \begin{eqnarray*}
\frac{\mathrm{d}D_{N,i}(t)}{\mathrm{d}t} &=& - (\lambda _i + \mu _i + \eta ) D_{N,i}(t) + 2 \lambda _i D_{N,i}(t)E_i(t) \\
+\, \sum \limits _{j\ne i}^{M} \left(\frac{\eta }{M-1} D_{N,j}(t) \right) \mbox{.}
\end{eqnarray*}

We derive the differential equation for $E_i(t)$ in a similar manner:


(4) \begin{eqnarray*}
\frac{\mathrm{d}E_i(t)}{\mathrm{d}t} &=& \mu _i - \left(\lambda _i + \mu _i + \eta \right)E_i(t) + \lambda _i E_i(t)^2 \\
+\, \sum \limits _{j\ne i}^{M} \left(\frac{\eta }{M-1} E_j(t) \right)\mbox{.}
\end{eqnarray*}

Because these differential equations cannot be solved analytically, we solve them numerically using a postorder (tip-to-root) algorithm that moves backwards in time along each lineage. When we encounter an internal node—a speciation event where an ancestral lineage *X* gives rise to a left (*Y*) and a right (*Z*) daughter lineage—we calculate the initial subtree probabilities as ${D_{X,i}(t) = D_{Y,i}(t) \times D_{Z,i}(t) \times \lambda _i}$, where $\lambda _i$ is the speciation rate given that the process is in rate category *i*. We continue this procedure until reaching the root of the tree, where $D_{R,i}(t_{R})$ is the probability of observing the entire study tree under the BDS process, given that the root is in rate category *i* (i.e., the conditional likelihood). To calculate the likelihood of the tree under the BDS process, we compute the weighted average of the *M* conditional likelihoods:


(5) \begin{eqnarray*}
L(\theta _\lambda , \sigma _\lambda , \theta _\mu , \sigma _\mu , \eta , \rho \mid \Psi ) = \sum _{i=1}^M D_{R,i}(t_{R}) \pi _i,
\end{eqnarray*}

where $\pi _i$ is the prior probability that the root is in rate category *i*. For simplicity, we assume that all rate categories are equally likely a priori (${\pi _i = 1 / M}$). However, other nonuniform prior probabilities on the root category could be used when warranted (see Section S2.2 of the Supplementary Material). Finally, we can condition the process on survival (i.e., that both daughters of the root leave surviving and sampled descendants):


(6) \begin{eqnarray*}
L(\theta _\lambda , \sigma _\lambda , \theta _\mu , \sigma _\mu , \eta , \rho \mid \Psi , \mathrm{survival})\\
\quad = \frac{L(\theta _\lambda , \sigma _\lambda , \theta _\mu , \sigma _\mu , \eta , \rho \mid \Psi )}{1 - \sum _{i = 1}^M E_i(t_{R})^2 \pi _i}.
\end{eqnarray*}

Note that we specify a marginal condition on survival where we compute the probability of survival summed over all diversification-rate categories. As a generative process, this marginal condition implies that if a realization/simulation of the process did not survive, then we redraw the root diversification-rate categories and repeat the simulation. Other approaches that use data augmentation (e.g., Maliet and Morlon 2022; Quintero et al. 2024) are likely to perform a joint condition instead (for a related discussion on the impact of marginal vs. joint conditioning, see Capobianco and Höhna 2025).

### Mapping Diversification Histories Over the Tree

The approach described in the previous section to numerically solve the differential equations ${\mathrm{d}D_{N,i}(t)} / {\mathrm{d}t}$ and ${\mathrm{d}E_i(t)} / {\mathrm{d}t}$ from the tips to the root of the tree allows us to compute the probability of realizing the study tree under the BDS process. The probability that we calculate accounts for all possible histories of diversification rate-shift events and all possible diversification-rate categories for each branch. In doing so, therefore, our algorithm sums over (“integrates out”) the quantity of biological interest: the diversification rates of each branch of the tree. Accordingly, we developed an approach to estimate the branch-specific rates of speciation and extinction by mapping diversification histories over the study tree. Our approach is similar in spirit to stochastic-mapping approaches that map character histories over the branches of a phylogeny (e.g., Nielsen 2002). Under stochastic character mapping, character histories are simulated in a preorder (root-to-tip) traversal of the tree, where each history specifies the number and location of character-state changes, and also paints the character state on each branch of the tree. Our approach is similar, but involves simulating diversification rate histories rather than character histories over the tree (Freyman and Höhna 2019). Each diversification history specifies the number and location of diversification-rate shifts, and also paints the “state” of the branching process (i.e., the diversification-rate category) on each branch of the tree.

**Figure 4.**
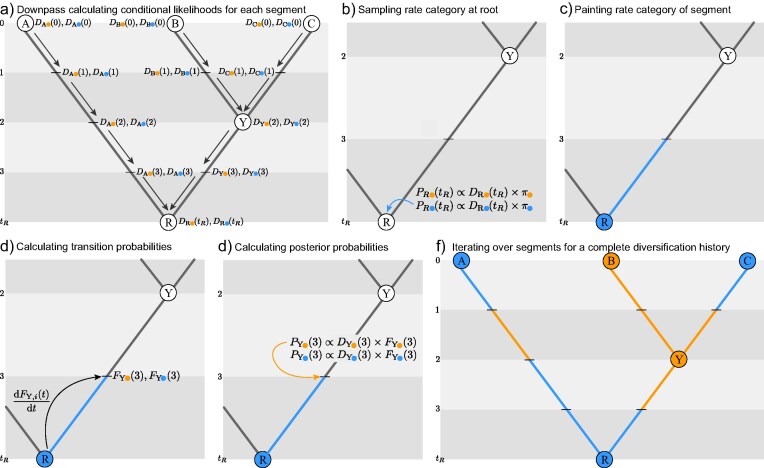
Mapping diversification histories. A hypothetical tree with three lineages, where the BDS process involves shifts among two diversification-rate categories (orange and blue). For computational tractability, we discretize each branch length into a number of time segments; for clarity, segments are defined by three fixed time points here. a) First, we perform a tip-to-root traversal of the tree, sequentially visiting each segment of each branch, calculating the vector of conditional likelihoods for each rate category, $D_{N,i}(t)$, that is, the probability that lineage *N* in rate category *i* at some point in the past, *t*, gives rise to the observed lineage. b) At the root, we initialize a root-to-tip traversal of the tree by sampling a rate category proportional to its posterior probabilities, $P_{R,i}(t_{R})\propto D_{R,i}(t_{R})\times \pi _i$, where $\pi _i$ is the prior probability of rate category *i*. c) We then move along the first branch segment, and paint it with the sampled rate category. d) Next, we calculate the transition probabilities, $F_{N,i}(\mathrm{end})$, for example, the probability that we transition from the selected rate category at the root to each of the *i* rate categories at the end of the first branch segment. e) We then calculate the posterior probabilities for the rate category at the end of the branch segment, $P_{N,i}(t_{\mathrm{end}})\propto D_{N,i}(t_{\mathrm{end}})\times F_{N,i}(t_{\mathrm{end}})$ and sample a rate category proportional to its posterior probabilities to initialize the rate categories at the beginning of the next segment. f) We iterate this process segment-wise until we reach the tips of the tree to provide a complete diversification history.

In principle, we expect that rate shifts may occur at any point along a branch. For computational tractability, we discretize each branch into a large but finite number of segments, for example, 5000 segments from root to tip. Our algorithm begins with a postorder tip-to-root traversal that computes the conditional likelihood of the data, $D_{N,i}$, at the end of each segment on each branch (Fig. 4a). We then perform a preorder traversal to simulate the history along each branch. We begin by selecting the initial rate category at the root of the tree by sampling it in proportion to its posterior probability (Fig. 4b):


\begin{eqnarray*}
P_{R,i}(t_{R}) = \frac{D_{R,i}(t_{R})\times \pi _i}{\sum \limits _{j\!=\!1}^{M} D_{R}(t_{R})\times \pi _{j}}.
\end{eqnarray*}

We begin by moving from the root to the present along one of its daughter branches and “paint” the first segment of that branch according to the rate category sampled at the root (Fig. 4c). Next, our goal is to sample the rate category at the end of the first segment. This involves three main steps: 1) we first compute the probability of ending in rate category *j* at the end of the segment (i.e., the transition probability from state *i* to state *j*) for all rate categories *j*; 2) we then compute the posterior probability for each rate category at the end of the segment, and 3) finally, we sample a rate category at the end of the segment in proportion to the posterior probabilities.

Computing the transition probability from category *i* to category *j* over a branch segment involves solving a set of differential equations (Fig. 4d). We denote the probability that lineage *N* is in rate category *i* at time *t* as $F_{N,i}(t)$. We initialize these probabilities based on the rate category sampled at the beginning of the segment, that is, ${F_{N,i}(t_{\mathrm{begin}}) = 1}$ and the ${M-1}$ unsampled rate categories have probability ${F_{N,j \ne i}(t_{\mathrm{begin}}) = 0}$. We calculate the change in these probabilities by considering all of the events that could occur in tiny time steps of size $\Delta t$ along the branch segment toward the present. These events are the same as those for the conditional likelihoods, giving rise to the set of difference equations:


(7) \begin{eqnarray*}
F_{N,i}(t-\Delta t) &= & (1-\lambda _i \Delta t - \mu _i \Delta t - \eta \Delta t) \times F_{N,i}(t) \times \quad \mathrm{(A)} \\
+\, \lambda _i \Delta t \times F_{N,i}(t) \times E_i(t-\Delta t) \quad \mathrm{(B)} \\
+\, \lambda _i \Delta t \times F_{N,i}(t) \times E_i(t-\Delta t) \quad \mathrm{(C)} \\
+\, \sum _{j \ne i}^{M} \frac{\eta \Delta t}{M-1} F_{N,j}(t),\quad \mathrm{(D)}
\end{eqnarray*}

where the letters for the terms correspond to those in Figure 3. The corresponding differential equation is


(8) \begin{eqnarray*}
\frac{\mathrm{d}F_{N,i}(t)}{\mathrm{d}t} &= & - (\lambda _i + \mu _i + \eta ) F_{N,i}(t) + 2 \lambda _i F_{N,i}(t) E_{i}(t)\\
+\, \sum \limits _{j\ne i}^{M} \frac{\eta }{M-1} F_{N,j}(t).
\end{eqnarray*}

Note that these equations are similar to equations (2) and (3), but here we are moving forward in time. We compute the probabilities of the state at the end of the segment at time $t_{\mathrm{end}}$, $F_{N,i}(t_{\mathrm{end}})$, by solving these differential equations given the initial probabilities $F_{N,i}(t_{\mathrm{begin}})$. Note that, because $F_{N,i}(t)$ depends on $E_i(t)$, we must simultaneously solve $E_i(t)$ forward in time as well (starting from the initial probability $E_{i}(t_{\mathrm{begin}})$ at the beginning of the segment).

We have now computed the probability of transitioning to each rate category at the end of the segment, given the rate category at the beginning of the segment. The posterior probability of each rate category at the end of a segment is the probability of transitioning into that rate category, and then subsequently giving rise to the observed data (the subtree between the end of the segment and the present; Fig. 4e). Accordingly, the posterior probability is


\begin{eqnarray*}
P_{N,i}(t_{\mathrm{end}}) = \frac{D_{N,i}(t_{\mathrm{end}}) \times F_{N,i}(t_{\mathrm{end}})}{\sum \limits _{j\!=\!1}^{M} D_{N,j}(t_{\mathrm{end}}) \times F_{N,j}(t_{\mathrm{end}})}.
\end{eqnarray*}

We then sample a rate category at the end of the segment in proportion to these posterior probabilities (Fig. 4e). We initialize the probabilities at the beginning of the next segment, $F_{N,i}(t_{\mathrm{end}})$, based on the sampled rate category, and paint it according to the sampled rate category.

We iterate this procedure for each segment until we reach the end of the current branch (Fig. 4f). If the branch ends at a speciation event (i.e., an internal node), the initial probabilities for the first segment of each daughter branch are set according to the sampled diversification-rate category at the end of the current branch. We then repeat this procedure for each branch until we reach the tips of the tree, resulting in a complete map of the diversification-rate history over the tree. This history specifies the number and location of all diversification-rate shifts, and the diversification rate of each branch segment.

### Estimating Parameters of the BDS Process

We estimate parameters of the BDS process in a Bayesian statistical framework. The posterior probability distribution of the BDS process parameters is


(9) \begin{eqnarray*}
\overbrace{P(\theta _\lambda , \sigma _\lambda , \theta _\mu , \sigma _\mu , \eta \mid \Psi , \rho )}^{\text{joint posterior distribution}} & \propto & \\
& & \underbrace{P(\Psi \mid \theta _\lambda , \sigma _\lambda , \theta _\mu , \sigma _\mu , \eta , \rho )}_{\mathrm{likelihood}} \\
& & \\
& & \qquad \qquad \qquad \times \\
& & \\
& & \underbrace{P(\theta _\lambda ) P(\sigma _\lambda ) P(\theta _\mu ) P(\sigma _\mu ) P(\eta )}_{\mathrm{priors}}, \\
\end{eqnarray*}

where $\Psi$ is the study tree and $\rho$ is the lineage-sampling fraction, and the BDS (hyper)parameters are listed below. Unless specified otherwise, we use the following prior distributions in all of our analyses:


\begin{eqnarray*}
\theta _\lambda & \sim & \mathrm{Uniform}(0, 100) \\
\sigma _\lambda & \sim & \mathrm{Uniform}(0, 5H) \\
\theta _\mu & \sim & \mathrm{Uniform}(0, 100) \\
\sigma _\mu & \sim & \mathrm{Uniform}(0, 5H) \\
\eta & \sim & \mathrm{Uniform}(0, \mathrm{TL}/100),
\end{eqnarray*}

where ${H\!=\!0.587405}$, such that 95% of the prior distribution spans one order of magnitude (Höhna et al. 2017), and TL is the tree length (sum of the branch lengths). We compute the likelihood using equation (5), and use Markov chain Monte Carlo sampling to estimate the joint posterior distribution of the BDS model parameters. Additionally, we simulate one diversification history using the stochastic-mapping algorithm (either every MCMC iteration or every *i*th iteration, if thinning is desired).

#### Summarizing branch-specific diversification rates

For each simulated history, we compute the average speciation, extinction, and net-diversification rate for each branch. Specifically, for a given simulated history, we calculate the rate as a weighted average, that is, weighted by the relative duration spent in each rate category, for each branch. This corresponds to a single sample from the joint posterior distribution of average branch-specific diversification rates. We repeat this procedure over the course of the MCMC to generate the joint posterior distribution of average branch-specific diversification rates. The branch-specific diversification rates can be visualized with the R package RevGadgets (Tribble et al. 2022).

### Implementation of the Birth–Death Process in RevBayes

We implemented the likelihood and stochastic-mapping algorithms in RevBayes (Höhna et al. 2016). Our implementation relies on the boost c++ library to numerically solve differential equations (3) and (4) to compute the likelihood. Specifically, we use the Runge–Kutta Dormand–Prince 5 method with default adaptive-error step size (Dormand and Prince 1980), as we found this to be the most efficient option.

Our stochastic-mapping implementation involves discretizing each branch into a large but finite number of segments. Specifically, we define a segment size of $h = t_{R} / L$, where $t_R$ is the age of root node, such that larger values of *L* correspond to smaller segments (default $L = 5000$). We then divide each branch into segments of size *h* with any remainder captured in a final segment of size ${<}h$. We then perform a postorder traversal of the tree solving equations (3) and (4) as in our likelihood calculation, this time storing conditional likelihoods at the end of each segment for each branch (Fig. 4a). We then perform a preorder traversal of the tree, sampling states and solving equation (8) and the coupled extinction probabilities in equation (4) along each segment again using the Runge–Kutta Dormand–Prince 5 method (Dormand and Prince 1980) with default adaptive error tolerances.

## Validating the BDS Method

We used a variety of numerical and analytical tools to validate the theory and implementation of our BDS method. Specifically, we 1) used analytical benchmarks for special cases of the BDS process; 2) compared inferences using our likelihood function with those of a mathematically equivalent alternative algorithm; and 3) performed a well-calibrated simulation to confirm that posterior distributions inferred under our BDS process have the correct statistical interpretation, that is, that the coverage probabilities are correct.

### Validating with Special Cases of the BDS Process

**Figure 5.**
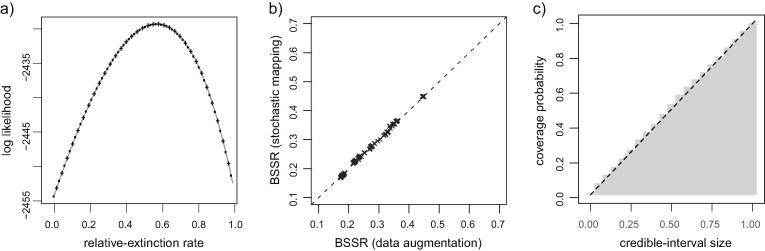
Validating the theory and implementation of the BDS method. a) We compared the analytical likelihoods under a constant-rate birth–death process (shaded line) over all relative-extinction rates with likelihoods estimated under a special case of our BDS process—that is, where the shift rate is 0 (${\eta = 0}$) and all *K* rate categories are identical (${\lambda _i=\lambda _j}$ and ${\mu _i=\mu _j}$)—using both the numerical-integration ($+$ symbols) and data-augmentation ($\circ$ symbols) methods. b) We compared branch-specific speciation rates (BSSR) inferred using the stochastic-mapping method with those using a mathematically equivalent data-augmentation method. Estimates based on these two independent methods are identical up to Monte Carlo sampling errors. c) Coverage probabilities are correct. Figures a) and b) are based on analyses of the primate phylogeny from Springer et al. (2012), whereas Figure c) is based on analyses of 1000 simulated trees.

The ability to correctly calculate the likelihood of a study tree under a given diversification model is fundamental to reliable inference. Some previous methods for inferring lineage-specific diversification-rate variation—such as BAMM (Rabosky 2014) and MEDUSA (Alfaro et al. 2009)—have been shown to calculate likelihoods incorrectly (May and Moore 2016; Moore et al. 2016). It is therefore paramount to confirm that the likelihoods calculated under our BDS process are correct. Ideally, we would validate our numerically estimated likelihoods using analytical results. Although analytical results are available for the constant-rate birth–death process, they are not available for the BDS process. It is nevertheless possible to collapse the BDS process to a constant-rate birth–death process. Specifically, the BDS process simplifies to a constant-rate birth–death process when the shift rate is set to zero (${\eta = 0}$), and the diversification rates are identical for each rate category (${\lambda _i=\lambda _j}$ and ${\mu _i=\mu _j}$). (Note that the latter restriction is necessary to specify the constant-rate birth–death process, as we would otherwise obtain a mixture of constant-rate birth–death processes.) The ability to analytically compute likelihoods under the constant-rate birth–death model provides a benchmark to confirm that our algorithm for computing likelihoods under the BDS process is correct when rates of diversification are constant across lineages. We performed experiments to confirm that analytical likelihoods under the constant-rate birth–death process are identical to the numerical likelihoods estimated under this restricted BDS process (Fig. 5a). We provide details of this validation experiment in Section S3.1.1 of the Supplementary Material.

We performed a second experiment to confirm that the BDS process correctly models diversification-rate shifts. Ideally, we would perform this validation using an analytical benchmark. We might expect that the Poisson distribution provides the appropriate analytical benchmark. That is, perhaps the distribution for the number of diversification-rate shifts, *S*, over the entire tree with length TL follows a Poisson distribution with rate $\Lambda = \eta \mathrm{ TL}$. Although the Poisson distribution correctly describes events that are conditionally independent of the tree (e.g., such as a trait that evolves passively over the tree), it will not correctly describe the distribution of events that change the process that is generating the tree (Moore et al. 2016). That is, shifts to a higher diversification rate will give rise to more lineages, and shifts to lower rates will shrink it. However, we could use this analytical benchmark to validate the number of diversification-rate shifts under a different restricted BDS process; that is, one that allows rate shifts (${\eta \ne 0}$), but specifies equal diversification rates for each category (${\lambda _i=\lambda _j}$ and ${\mu _i=\mu _j}$). Accordingly, under this restricted BDS process, the inferred posterior distribution for the number of “rate shifts” should be identical to its analytical Poisson distribution. Again, our experiments confirm that the inferred distribution of rate shifts under this restricted BDS process matches the analytical distribution (Supplementary Fig. S4). We provide details of this experiment in Section S3.1.2 of the Supplementary Material.

### Validating with a Second Algorithm

To validate our derivation and implementation of the likelihood function for the unrestricted BDS process where diversification rates are free to vary among lineages, we also developed a second, mathematically equivalent approach for calculating the likelihood of an observed tree under the BDS process based on “data augmentation.” In outline, this approach involves adding diversification history by specifying the number and location of rate shifts and the diversification rates for every branch of the study tree under the BDS process, and then calculating the likelihood of realizing the study tree under that diversification history. We use reversible-jump Markov chain Monte Carlo (Green 1995) to numerically integrate over diversification histories on observed lineages, where the histories are sampled in proportion to their probability under the BDS process. This is mathematically equivalent to integrating over all possible rate shifts on observed lineages by exhaustive enumeration (Fig. 5b). Because we cannot map diversification histories on unsampled and extinct lineages (cf. Maliet and Morlon 2022), we still use numerical integration (equation 3) to correctly compute extinction probabilities, $E_{i}(t)$. We provide details of the data-augmentation approach and reversible-jump MCMC algorithms in Section S1 of the Supplementary Material.

Because the stochastic-mapping and data-augmentation approaches are mathematically equivalent, branch-specific diversification rates inferred using these two independent methods should be identical if they are correctly derived and implemented. Indeed, our analyses of the primate phylogeny of Springer et al. (2012) using these two independent approaches inferred identical branch-specific diversification rates (Fig. 5b). We note, however, that the data-augmentation approach is $\approx$20- to 500-fold less computationally efficient than the stochastic-mapping approach (Supplementary Fig. S6). We provide details of this validation experiment in Section S3.2 of the Supplementary Material.

### Simulation-Based Calibration: Confirming Correct Coverage

In a Bayesian analysis, the $X\%$ posterior credible interval of a parameter should contain the true parameter value $X\%$ of the time (Cook et al. 2006; Talts et al. 2018; Mendes et al. 2025). When parameter values for simulations are drawn from their prior distributions, failure to achieve this expected relationship between credible interval size and coverage probability indicates that either the likelihood function and/or MCMC algorithm is implemented incorrectly. To validate our likelihood function and MCMC algorithms, we simulated 1000 trees each with 200 tips under the BDS model. We then performed inference on each simulated tree under the true model and confirmed that the credible intervals for the branch-specific net-diversification rates matched the expected coverage (Fig. 5c). We provide details of this validation experiment in Section S3.3 of the Supplementary Material.

## Characterizing the Statistical Behavior of the BDS Method

**Figure 6.**
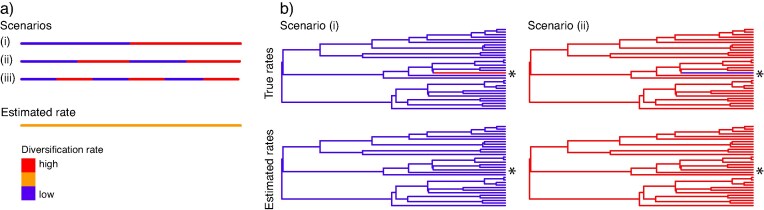
Some intrinsic and challenging properties of lineage-specific diversification processes. Lineage-specific diversification models, like the BDS process, are weakly identifiable with respect to the event-rate parameter, $\eta$, such that the number of diversification-rate shifts is highly sensitive to the choice of prior on $\eta$. a) Hypothetical scenarios depicting rate-shift events across a single branch, where the prior for the event-rate parameter, $\eta$, specifies a low, moderate, or high number of diversification-rate shifts (Scenarios i–iii, respectively). This prior-sensitivity issue clearly limits our ability to estimate the number and location of diversification-rate shifts; however, in principle, it does not impact our ability to reliably estimate the average branch-specific diversification rate. b) The statistical power of lineage-specific diversification models scales with the size of the subtree subtending a rate shift: rate shifts along terminal branches cannot be estimated. Accordingly, when a shift to a higher rate occurs on a terminal branch, we will underestimate the true diversification rate (Scenario i). Similarly, when a shift to a lower rate occurs on a terminal branch, we will overestimate the true diversification rate (Scenario ii). This statistical-power issue, in turn, is expected to cause us to underestimate the variance in diversification rates across the tree (i.e., to underestimate parameters $\sigma _\lambda$ and $\sigma _\mu$ of the BDS process).

Given the complexity of the BDS process, even a theoretically correct and properly implemented method will have inference limitations. For example, the number and location of rate shifts are weakly identifiable. That is, a given tree is equally likely to result from a diversification history with many relatively small shifts, or a history with fewer relatively large shifts (Moore et al. 2016). Accordingly, we expect the number of rate shifts across a given branch to be highly sensitive to the assumed prior on the rate of shift events, $\eta$. In principle, however, it nevertheless remains possible to reliably estimate the average branch-specific diversification rates under the BDS process, irrespective of the number, size, or nature of rate shifts (Fig. 6a).

A second challenge for estimating lineage-specific diversification rates is related to statistical power. Specifically, the power to infer lineage-specific diversification rates decreases with the size of the subtree subtending a rate shift (e.g., Moore et al. 2004), with no power to infer diversification-rate shifts that are subtended by a single lineage (i.e., that occur along terminal branches). As a terminal lineage provides no additional information (i.e., no additional speciation events), the most likely scenario is that of no shift. Thus, the BDS process will give higher likelihoods to no shift events (and penalize additional shifts) on terminal branches, effectively leading to reduced power to estimate shifts on terminal branches. This is particularly sobering, given that a tree with *S* lineages has $2S-2$ branches; that is, about half are terminal branches. Accordingly, when a terminal branch experiences a shift to a higher rate, we will underestimate its true diversification rate. Similarly, when a terminal branch experiences a shift to a lower rate, we will overestimate its true diversification rate (Fig. 6b). We expect this inherent limitation—overestimating low diversification rates of terminal branches and underestimating high diversification rates on terminal branches—in turn, will cause us to underestimate the variance in diversification rates across the tree, that is, to underestimate the standard deviations of the lognormal distributions for the speciation and extinction rates (the parameters $\sigma _\lambda$ and $\sigma _\mu$, respectively).

We performed a simulation study to explore the statistical behavior of our BDS method. Specifically, we simulated 100 trees under the BDS process with parameters specified to generate trees spanning a wide range of sizes (filtering trees with $\le 20$ and $\ge 10,000$ tips), where each tree had a moderate number of diversification-rate shifts of variable magnitude (Supplementary Figs. S9 and S10). We then performed analyses of each simulated tree under the BDS process. We might have performed these analyses by specifying the parameters of the inference model to be identical to those of the true model (the model used to simulate the trees). However, this best-case inference scenario would tend to portray the behavior of our method in a favorable light. That is, when our method is applied to empirical data sets, we will not know the true process that gave rise to a given study tree. We can therefore obtain a more meaningful characterization of our method by using a somewhat misspecified inference model. Accordingly, we specified the parameters of inference model to depart substantially from those of the generating model; that is, the distributions for the diversification-rate parameters, $\lambda$ and $\mu$, and for the rate-shift parameter, $\eta$, were much more diffuse than those of the true model, and the number of rate categories used for inference differed from that used in simulation ($K = 6$ vs. 4 rate categories, respectively). We provide a more detailed description of our simulation study in Section S4.1 of the Supplementary Material.

**Figure 7.**
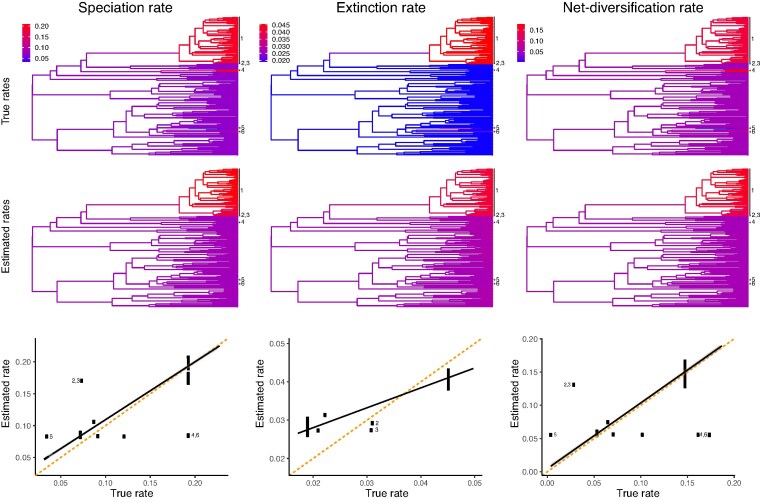
Exploring the ability to infer branch-specific diversification rates in a single simulated tree. The top row depicts a single simulated tree (#35) with branch colors indicating the true speciation rate (left), extinction rate (center), or net-diversification rate (right). The second row depicts the same tree with branch colors indicating estimates of the corresponding rates. Numbers across the tips of the trees indicate lineages with distinct (true) diversification rates. The bottom row plots the true rate against the estimated rate for each parameter, where each point corresponds to a single branch in the tree, and the black line is the regression fit to those points (the dashed line indicates perfect estimation; estimated rate = true rate). This simulated history includes a single instance where a rate shift is subtended by a large subtree (1) and several shifts that have one (or a few) descendant lineages (2–6). Encouragingly, branch-specific diversification rates are estimated very well for the large subtree. By contrast, shifts subtended by small subtrees are poorly estimated as expected, shifts to lower rates (2, 3, and 5) are overestimated, whereas shifts to higher rates (4 and 6) are underestimated (cf. Fig. 6b).

We begin by exploring the results of our analyses for a single simulated tree (Fig. 7). This realization of the BDS process—simulated tree number 35—is typical of those used in our simulation study: It has 221 lineages (on average, our simulated trees had 379 lineages) and experienced six diversification-rate shifts. A single diversification-rate shift was subtended by a relatively large subtree with 74 lineages, whereas the others were subtended by small subtrees with one to three lineages. For the shift subtended by a large subtree, branch-specific diversification rates are well estimated: the inferred speciation, extinction, and net-diversification rates for each branch are nearly identical to the corresponding true branch rates. By contrast, branch-specific diversification rates are poorly estimated in cases where shifts are subtended by small subtrees. In these cases, shifts to lower rates are overestimated, and shifts to higher rates are underestimated.

**Figure 8.**
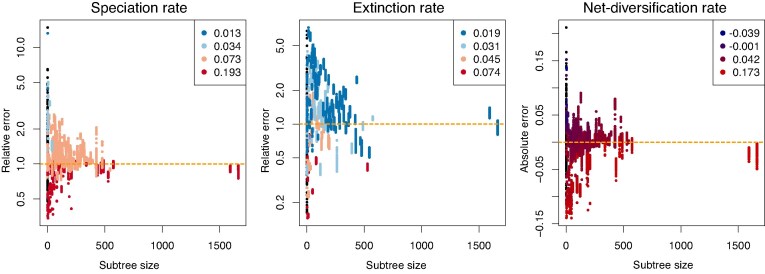
Exploring the ability to estimate branch-specific diversification rates for all simulated trees. Each panel summarizes the results of our simulation study for a different branch-specific rate parameter: speciation rate (left), extinction rate (center), and net-diversification rate (right). Each dot represents the posterior mean estimate of a single branch in one of the 100 simulated trees. The color of each dot corresponds to the true (simulated) rate for that branch, demonstrating the impact of the rate category on the inferred bias. The *x*-axis plots the size of the subtree subtending a rate-shift event, excluding all subtrees with additional rate shifts. Note that all branches belonging to that clade receive the same subtree size (*x*-axis) and represent the size of the clade that experienced the shift (minus all lineages for a nested shift event). The *y*-axes plot the relative error ($\epsilon = \mathrm{ estimated}\div \mathrm{ true}$) for estimates of speciation and extinction rates, and the absolute error ($\epsilon = \mathrm{ estimated}-\mathrm{ true}$) for estimates of net-diversification rates. The dashed line indicates perfect estimation (zero relative or absolute error). Overall, rate estimates are unbiased (error is symmetric about the dashed line). As expected, error in branch-specific diversification rates is highest for rate shifts subtended by a single lineage and decreases for subtrees of increasing size. Also, as expected, when the subtrees’ subtending rate shifts are small, the method tends to underestimate higher rates and to overestimate lower rates (cf. Fig. 6b).

The behavior of our BDS method for this single tree is similar to that across the sample of 100 simulated trees (Fig. 8). Specifically, our results confirm that the power to estimate branch-specific diversification rates under the BDS process scales with the size of the subtree subtending diversification-rate shifts. The estimation error is inversely related to the size of the subtree subtending a rate shift. When the subtrees’ subtending rate shifts are small, the method tends to underestimate higher rates and to overestimate lower rates (cf. Fig. 6b). Importantly, the results of our simulation study indicate that estimates of branch-specific diversification rates under the BDS process are unbiased.

In summary, our simulation study indicates that our BDS method exhibits the expected statistical behavior. Across our simulated trees, we observed four patterns: 1) if a simulated tree did not experience a diversification rate shift, then our method does not infer variation in branch-specific diversification rates, indicating a low false-positive rate (Supplementary Fig. S13); 2) if a simulated tree experienced one or more rate shifts subtended by a sufficiently large subtree, then our method accurately estimates branch-specific diversification rates (Fig. 7); 3) if a simulated tree was small (e.g., with $\le 25$ tips), then our method is unable to accurately estimate branch-specific diversification rates, and may even produce biased estimates (Supplementary Fig. S15); and 4) if a simulated tree experienced diversification rate shifts that gave rise to very small subtrees, then our method does not infer variation in branch-specific diversification rates (Supplementary Fig. S16). Overall, these simulations indicate that our method provides accurate and unbiased estimates of branch-specific diversification rates given sufficient data.

## Empirical Analyses of Primates

We performed a series of analyses on a time-calibrated phylogeny of 367 primate lineages (Springer et al. 2012) with three main goals: 1) to demonstrate how we can use Bayes factors to select among competing lineage-diversification models; 2) to assess the impact of discretization bias on estimates of branch-specific diversification rates; and 3) to explore the impact of prior sensitivity of the shift-rate prior, $\eta$, on estimates of branch-specific diversification rates. We detail these analyses in Section S4 of the Supplementary Material.

### Comparing the Constant-Rate and BDS Models

Our approach allows biologists to compare the relative fit of alternative diversification models with a given study tree. For example, a sensible first step in any study of diversification rates is to assess whether the use of a variable-rate diversification model is warranted. That is, we are asking the question: “Is our study phylogeny significantly more likely to be the result of a constant-rate or a variable-rate diversification process?” We can address this question by comparing the relative fit of two candidate diversification models to a given data set by calculating the corresponding Bayes factor (Kass and Raftery 1995):


\begin{eqnarray*}
\mathrm{ BF}_{VC} = \frac{f_V(\Psi )}{f_C(\Psi )},
\end{eqnarray*}

where the inferred study tree, $\Psi$, is treated as an observation, and $f_C(\Psi )$ and $f_V(\Psi )$ are the marginal likelihoods under the constant-rate and BDS models, respectively. Note that we could accommodate phylogenetic uncertainty by instead treating the alignment of DNA sequences, ${\mathbf {X}}$, as our observations (Barido-Sottani and Morlon 2023), in which case the marginal likelihoods, $f_C({\mathbf {X}})$ and $f_V({\mathbf {X}})$, would be averaged over the posterior distribution of time-calibrated trees.

To assess whether our primate study tree is the outcome of a constant-rate or BDS process, we first estimated the marginal likelihoods for these two models using the path-sampling algorithms implemented in RevBayes (Lartillot and Philippe 2006; Xie et al. 2011; Höhna et al. 2021). We then used these marginal-likelihood estimates to compute Bayes factors for the competing models, which indicates a clear preference for the BDS model; that is, where $\log _e \mathrm{ BF}_{VC} = 10.8$ is interpreted as “decisive” (Jeffreys 1939), “extreme” (Lee and Wagenmakers 2014), or “very strong” (Kass and Raftery 1995) support for the BDS model over the constant-rate model.

### Branch-Specific Diversification-Rate Estimates Are Robust to Discretization Bias

Our BDS process approximates the continuous distributions for the speciation and extinction rates with *K* discrete rate categories. The quality of the approximation depends on the chosen number of rate categories (Fig. 1): using too few rate categories will provide an inadequate approximation of the continuous distributions, which may bias estimates of the branch-specific diversification rates. Conversely, increasing the number of rate categories increases the computational burden: using more than the required number of categories will needlessly increase the time required to perform our analyses. Because the number of rate categories is not a parameter of our BDS process, we cannot use model-selection procedures, such as Bayes factors, to select an appropriate value for *K*. Here, we demonstrate a protocol for selecting an adequate number of rate categories.

**Figure 9.**
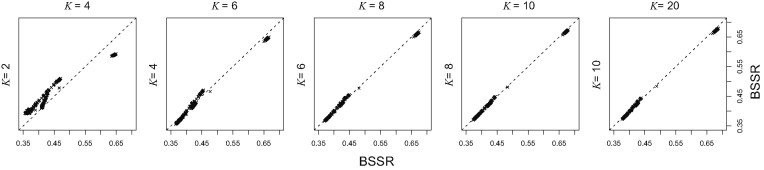
Comparing BSSR estimates for different numbers of rate categories. We estimated the posterior mean BSSR for each branch of the primate tree under a series of BDS analyses that incrementally increased the number of rate categories, ${K = (2, 4, 6, 8, 10, 20)}$, that were used to approximate the continuous distributions for the speciation and extinction rates. We then compared the resulting BSSR estimates for adjacent values of *K*. The impact of discretization bias is evident for analyses using very small numbers of rate categories; that is, the BSSR estimates are quite different for adjacent values when *K* is small (left panel). However, as the number of rate categories increases, the BSSR estimates converge to stable values. For this data set, $K\ge 6$ discrete categories appears to provide an adequate approximation.

We explored the impact of discretization bias on estimates of the BSSRs by performing a series of analyses that incremented the number of rate categories, ${K = \lbrace 2, 4, 6, 8, 10, 20 \rbrace }$, while holding all other settings constant. As expected, for a very small number of rate categories, BSSR estimates exhibit discretization bias (Fig. 9, left panels). Encouragingly, as the number of rate categories increases, the BSSR estimates converge to stable values (Fig. 9, center-right panels). For the primate data set, six rate categories appear to provide a sufficient approximation of the underlying continuous base distribution. These results suggest that an adequate approximation of the continuous process can be achieved with a modest and computationally tractable number of rate categories, and our experiment also suggests a viable protocol for objectively selecting an adequate number of rate categories for a given empirical data set.

### Branch-Specific Diversification-Rate Estimates Are Robust to the Event-Rate Prior

As we have discussed, lineage-specific diversification models, including our BDS process, confound estimates of the number of diversification-rate shifts. That is, posterior estimates of the number of diversification-rate shifts are very sensitive to the assumed prior on the rate of shift events, $\eta$ (Moore et al. 2016). This might raise concerns that other parameters, particularly estimates of the branch-specific diversification rates, are similarly sensitive to the choice of event-rate prior. We performed analyses of the primate tree using a range of priors on $\eta$ that specified a corresponding range of values on the expected number of rate shifts, $E(S) = (1, 10, 20)$, while holding all other settings constant. For each event-rate prior, we estimated the posterior distribution for the number of rate shifts and the BSSRs.

**Figure 10.**
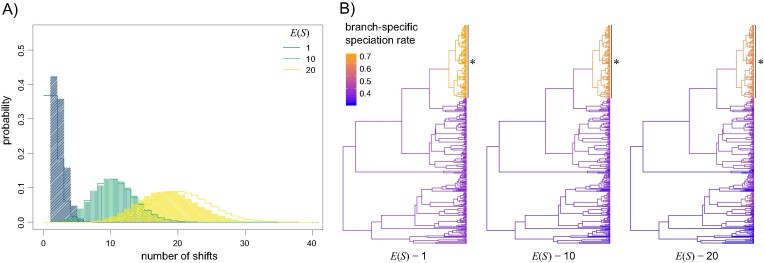
(In)sensitivity of estimates to the assumed prior on the number of diversification-rate shifts. We performed analyses of the primate tree under the BDS process using three different event-rate priors, $\eta$, such that the expected number of rate shifts, $E(S)$, was either 1, 10, or 20. a) Estimates of the number of rate shifts are highly sensitive to the assumed event-rate prior (i.e., the posterior and prior distributions are nearly identical). b) By contrast, estimates of the BSSRs are insensitive to the assumed event-rate prior: We consistently infer an elevated speciation rate in Old-World monkeys (asterisk) regardless of the assumed event-rate prior. Branch colors reflect the mean BSSR estimates; the scale bar is the same for all prior settings.

As expected, there is little information to estimate the number of rate shifts: inferred posterior distributions for the number of diversification-rate shifts were very similar to their corresponding prior distributions (Fig. 10a). By contrast, posterior estimates of the BSSRs are relatively insensitive to the choice of event-rate prior: BSSR estimates are very similar over a range of priors on $\eta$, and consistently indicate elevated speciation rates in the Old-World monkeys (Fig. 10b).

## Discussion

We have developed a method based on the BDS process that provides a general approach for the study of lineage-specific diversification-rate variation.

### Correctness of the BDS Method

Reliable inferences of lineage-specific diversification rates require a statistically coherent model that provides a mathematically sufficient description of the generative process. Our BDS process includes three types of events: speciation, extinction, and diversification-rate shifts. Diversification models are a type of Markov process where the probability of the next event depends exclusively on the current state (e.g., the diversification parameter values) of the process. Specifically, our BDS implementation allows for both rate-shift events at observed and unobserved (i.e., extinct or unsampled) lineages.

Accommodating diversification-rate shifts along extinct and/or unsampled lineages is not only a theoretical requirement of statistically coherent lineage-specific diversification models, it is also biologically sensible. Empirical phylogenies contain a small sample of extant lineages. Sampling is further reduced by extinction, as the vast majority of species that have existed are now extinct. For this reason, even rare phylogenies that contain a nearly complete sample of extant species are nevertheless likely to exclude a large fraction of their member species. Accordingly, our BDS process provides both a statistically coherent and biologically sensible model for inferring lineage-specific diversification-rate variation.

We performed a number of experiments to demonstrate that 1) likelihoods estimated under a restricted BDS process without diversification-rate shifts are identical to the corresponding analytical solutions under the constant-rate birth–death process (Fig. 5a); 2) the number of diversification-rate shifts estimated under a restricted BDS process with identical diversification-rate categories are identical to the corresponding analytical distribution (Fig. 5b); 3) our two mathematically equivalent approaches, the stochastic-mapping and data-augmentation methods, provide identical estimates of branch-specific diversification rates (Supplementary Fig. S4); and 4) credible intervals for branch-specific diversification-rate estimates exhibit the correct coverage (Fig. 5c). The results of these experiments indicate that our BDS method is implemented correctly.

We performed additional experiments to explore the behavior of our BDS method. To correctly and practically compute extinction probabilities while accommodating diversification-rate shifts on extinct and/or unsampled lineages, our method approximates the continuous distributions for the diversification rates with a finite number of discrete diversification-rate categories. Our analyses confirm that the continuous distributions can be adequately approximated with a small and computationally tractable number of rate categories (Fig. 9). Lineage-specific models are prone to prior sensitivity regarding the number of diversification-rate shifts (Moore et al. 2016). Nevertheless, we have demonstrated that posterior estimates of the focal quantities, the branch-specific diversification rates, under our BDS model are robust to the assumed prior on the rate of shift events (Fig. 10b). Finally, our simulation study confirms that branch-specific diversification rates are reliably estimated under our BDS method given 1) sufficient evidence of diversification-rate variation (i.e., where the study tree is reasonably large and a rate shift impacts a relatively large number of descendant lineages) and 2) that the BDS model provides a good fit to the study tree (Supplementary Figs. S11–S14). Accordingly, the battery of validation experiments that we preformed provides compelling evidence that the theory, implementation, and statistical behavior of our BDS method are correct.

### Generality of the BDS Method

Our BDS method expands our ability to study lineage-specific diversification-rate variation because it 1) is less biologically restrictive relative to other lineage-specific models; 2) allows specification of a variety of lineage-specific diversification models; and 3) may be applied under different statistical-inference scenarios. A recent study by Martínez-Gómez et al. (2024) has applied various approaches for estimating lineage-specific diversification-rates, including our approach described here. The tested approaches inferred different lineage-specific diversification-rates, with more similar approaches yielding more similar estimates.

#### Biological assumptions

Many events may cause a shift in rates of lineage diversification, including character evolution (e.g., changes in morphological, genomic, behavioral, or physiological features), biogeographic events (e.g., dispersal to a new area), the onset of a novel ecological association, abiotic/climatic changes, etc. In principle, these causal events may occur at any point in the evolutionary history of a lineage, that is, along any point of any branch and/or at any speciation event in our study phylogeny. When a diversification-rate shift occurs, it will impact all of the lineages descendant from that point in the tree. Our BDS model corresponds exactly to this generative process, where rate shifts occur along lineages rather than restricting rate shifts to speciation events.

By contrast, the cladogenetic model of Maliet et al. (2019) assumes that diversification-rate shifts occur exclusively at speciation events (see also Maliet and Morlon 2022). Accordingly, our BDS model is somewhat less restrictive with respect to its underlying biological assumptions compared with the cladogenetic model. It remains an open and interesting question whether the BDS or cladogenetic models generally provide a better description of the lineage-specific diversification-rate variation in real data sets. Fortunately, this question could be readily answered by simply performing model comparisons on a sample of empirical data sets to assess whether real trees are more likely to be the outcome of either lineage-specific process.

Our BDS process and the lineage-specific model described by Barido-Sottani et al. (2020) make identical biological assumptions. The primary difference between these two approaches centers on how they model discrete diversification-rate categories. Our BDS method uses a fixed number of rate categories, *K*, where *K* is set to a number that adequately approximates the corresponding continuous distributions (Fig. 9). The set of *K* diversification-rate values is simply the median of each of the *K* quantiles of the discretized distribution (Fig. 1). By contrast, the mixture model of Barido-Sottani et al. (2020) estimates both the number of rate categories and the value of each category. These seemingly minor differences in model design can nevertheless have large practical consequences: These two methods may infer qualitatively different patterns of diversification-rate variation when applied to empirical data sets (Martínez-Gómez et al. 2024). Again, it remains to be seen which model better describes the process of lineage-specific diversification-rate variation in real data sets.

#### Model diversity

To simplify our presentation, we focused on a single BDS model. In our BDS model, diversification-rate shifts involve a change in speciation and extinction rates, and we describe the diversification-rate parameters with a lognormal distribution with corresponding hyperparameters. However, our implementation of the BDS model in RevBayes provides tremendous flexibility in specifying a diversity of lineage-specific models. For example, we can specify BDS models where rate-shift events impact 1) exclusively speciation rates; 2) exclusively extinction rates; 3) both speciation and extinction rates; or 4) net-diversification rates (speciation–extinction). Moreover, for any of these BDS models, we can specify a wide range of distributions and corresponding parameters. For example, here we focused our discussion on lognormal distributions, but our implementation allows specification of many different (hyper)parameterizations, for example, with gamma or exponential distributions, to appropriately capture the nature of diversification-rate variation across lineages in a given data set.

Providing a diverse family of BDS models will facilitate both parameter estimation and hypothesis testing. That is, a larger pool of candidate models improves our chances of identifying an adequate inference model, that is, one capable of describing the process that gave rise to a specific data set. An adequate model provides a more reliable means for estimating branch-specific diversification rates. Importantly, the implementation of our method permits us to objectively choose among the diverse family of BDS models by computing Bayes factors. The ability to objectively assess the relative fit of a given data set to a large pool of candidate models provides greater scope for rigorously testing hypotheses about the nature of lineage diversification. That is, our ability to use Bayes factors to objectively select among competing diversification models is synonymous with testing competing hypotheses regarding the nature of diversification to assess whether diversification rates are constant or variable and, if variable, whether diversification-rate shifts tend to occur at speciation events or along lineages, etc.

#### Versatility of application

Studies of diversification-rate variation typically adopt a sequential-analysis approach (Höhna and Hsiang 2024). That is, divergence times are first inferred from an alignment of sequence data using a relaxed-clock model, the resulting posterior distribution of phylogenies is then summarized as some form of consensus tree, and the summary tree is treated as the phylogenetic “observations” that are used to estimate parameters of the assumed lineage-specific diversification model. Indeed, we used a sequential approach in our analyses of the primate data set, where the BDS process was used as the inference model to estimate branch-specific diversification rates from the primate summary tree.

There are several potential concerns with this sequential approach (Höhna and Hsiang 2024). First, treating the summary tree as an observation ignores underlying phylogenetic uncertainty in the topology and divergence-time estimates. Given that study trees are estimated from data, they are inherently uncertain. Accordingly, phylogenetic uncertainty should be accommodated in our estimates of lineage-specific diversification rates. Second, relaxed-clock models used to estimate divergence times include a tree prior to specify the prior distribution of topologies and speciation times. Specifically, the constant-rate birth–death process is often used as the tree prior for estimating divergence times. If diversification rates are truly variable across lineages of our study group, the misspecification of a constant-rate tree prior is apt to bias estimates of divergence times (Zhang et al. 2016; May et al. 2021; Barido-Sottani and Morlon 2023), which, in turn, may bias estimates of branch-specific diversification rates.

The implementation of our methods in RevBayes allows the BDS process to be applied in either a sequential or a joint-inference approach. In the latter approach, the BDS process is used as the tree prior, which allows us to simultaneously estimate the joint posterior distribution of dated phylogenies and branch-specific diversification rates. Adopting the BDS process in joint analyses offers several advantages. First, it correctly accommodates phylogenetic uncertainty because estimates of branch-specific diversification rates are marginalized over the posterior distribution of trees. Second, it may provide more accurate divergence-time estimates, which are often of direct biological interest (Barido-Sottani and Morlon 2023, 2025). Third, it avoids issues associated with using conflicting constant-rate prior models and variable-rate inference models.

## Conclusions

We have developed a method based on the BDS process that provides a general approach for the study of lineage-specific diversification-rate variation. Our approach and implementation in RevBayes includes a new approach to infer more efficiently branch-specific diversification rates: stochastic branch-rate mapping. Additionally, our general framework and implementation allow for many choices to tweak the model. This will enable model exploration and model comparison to better understand the process that generated historical biodiversity. We are optimistic that our BDS model will greatly enhance biologists’ ability to explore patterns of diversification in the Tree of Life.

## Supplementary Material

syag003_Supplemental_File

## Data Availability

All the original data and scripts necessary to repro- duce the analyses reported in this study can be accessed through the Dryad link: https://doi.org/10.5061/dryad.95x69p8t2.
