# Supplementary material for: Inferring Branch-Specific Rates of Lineage Diversification Under the Birth–Death-Shift Process
Source: Syst Biol. 2026 Jan 24;75(5):948–66. doi: 10.1093/sysbio/syag003 (PMC13445636; doi:10.1093/sysbio/syag003)
Supplement: Supplementary file 1 [file syag003_supplemental_file.pdf]

Inferring branch-specific rates of lineage diversification under the birth-death-shift process

Supplementary Material

Sebastian Höhna[1,2], William A. Freyman[3], Zachary Nolen[4], John P. Huelsenbeck[5], Michael R. May[5], Bruce Rannala[6], and Brian R. Moore[6]

[1] *Department of Earth and Environmental Sciences, Paleontology & Geobiology, Ludwig-Maximilians-Universität München, Richard-Wagner Straße 10, 80333 Munich, Germany*
[2] *GeoBio-Center, Ludwig-Maximilians-Universität München, Richard-Wagner Straße 10, 80333 Munich, Germany*
[3] *Department of Ecology, Evolution, and Behavior, University of Minnesota, Twin Cities 1479 Gortner Avenue, Saint Paul, MM 55108, U.S.A.*
[4] *Department of Biology, Lund University, Lund, Sweden*
[5] *Department of Integrative Biology, University of California, Berkeley 3060 VLSB #3140, Berkeley, CA 94720-3140, U.S.A.*
[6] *Department of Evolution and Ecology, University of California, Davis Storer Hall, One Shields Avenue, Davis, CA 95616, U.S.A.*

Contents

S1 The Data-Augmented Birth-Death-Shift Model . . . 3
S1.1 Proposal mechanisms for the data-augmentation method . . . 3
S2 BDS Model Parameterization . . . 5
S2.1 Alternative parameterizations of the speciation- and extinction-rate categories . . . 7
S2.1.1 Constant speciation and extinction rates . . . 7
S2.1.2 Lognormally distributed speciation and extinction rates with fixed hyperparameters . . . 8
S2.1.3 Alternative base distributions for speciation and extinction rates . . . 9
S2.1.4 Dependent rate categories . . . 10
S2.2 Root prior . . . 11
S2.3 Shift Rate . . . 11
S3 Validating the BDS Method . . . 12
S3.1 Validating with special cases of the BDS process . . . 12
S3.2 Validating with a second algorithm . . . 15
S3.3 Confirming correct coverage . . . 18
S4 Characterizing the Statistical Behavior of the BDS Method . . . 20
S4.1 Simulation Study: Testing the Accuracy of Branch-Specific Diversification-Rate Estimates . . . 20
S5 Empirical Analyses of Primates . . . 28
S5.1 Comparing lineage-diversification models using Bayes factors . . . 28
S5.2 Impact of discretization on branch-specific diversification-rate estimates . . . 29
S5.3 Impact of the event-rate prior on parameter estimates . . . 30
S5.4 Estimating hyperparameters of the BDS process . . . 32

S1 The Data-Augmented Birth-Death-Shift Model

The data-augmented model assumes the number, location, and parameters of the rate shifts on the study phylogeny are known (as augmented data). The probability density of the tree, $f(\Psi)$ (the augmented likelihood), is calculated in a manner similar to that of the full-marginalization approach described in the main text, except that the equation for the subtree probability is modified to account for the known locations of the rate-shift events,

$$\begin{aligned} D^{\mathrm{DA}}_{i,k}(t+\Delta t) =& (1-\mu_k\Delta t)\times(1-\eta\Delta t)\times \\ & \Big[(1-\lambda_k\Delta t)D^{\mathrm{DA}}_{i,k}(t) && (i) \\ & + (1-\eta\Delta t)\lambda_k\Delta t D^{\mathrm{DA}}_{i,k}(t)E_k(t) && (ii) \\ & + (1-\eta\Delta t)\lambda_k\Delta t D^{\mathrm{DA}}_{i,k}(t)E_k(t)\Big]. && (iii) \end{aligned} \tag{S1}$$

We use the superscript DA to distinguish the modified equation from its original version (*c.f.*, equations in main text). The resulting differential equation is

$$\frac{\mathrm{d}D^{\mathrm{DA}}_{i,k}(t)}{\mathrm{d}t} = -(\lambda_k+\mu_k+\eta)D^{\mathrm{DA}}_{i,k}(t) + 2\lambda_k D^{\mathrm{DA}}_{i,k}(t)E_k(t).$$

The extinction probability is unmodified, because we are not augmenting the extinct lineages with rate shifts. This is an important distinction, because if we would be able to compute the extinction probability analytically, then we also would be able to solve the full likelihood analytically [1]. We then use reversible-jump MCMC to add and remove rate-shifts to the tree, as described in the following subsection.

S1.1 Proposal mechanisms for the data-augmentation method

Our data-augmentation method involves augmenting the data (the study tree) with diversification histories that specify the number and location of rate shifts and the diversification rate of every branch of the phylogeny. We estimate the joint posterior probability distribution of the lineage-specific birth-death-shift parameters using reversible-jump MCMC to accommodate changes in the dimensionality of the model (associated with varying the number of diversification-rate shifts) over the tree. We add and remove events using birth and death moves, respectively. We begin by randomly selecting a birth or death move; either move is selected with equal probability $p=0.5$, except when the current number of diversification-rate shifts is zero, in which case we select a birth move with probability $p=1$. After selecting the move, we apply the corresponding algorithms to propose a new state of the Markov chain as described below.

Algorithm S1 Birth move

Inputs:
m: the current number of diversification-rate shifts.

$i \sim \text{Unif}(1, 2N-1)$ // randomly select a branch as the locus of the new diversification-rate shift
$s_{m+1} \sim \text{Unif}(0, t_i)$ // simulate the time of the new event on the selected branch, i, of length t
$\lambda_{m+1} \sim \text{Prior}$ // sample the speciation rates for the new event from the prior distributions
$\mu_{m+1} \sim \text{Prior}$ // sample the extinction rates for the new event from the prior distributions
$\lambda' = \{\lambda \cup \lambda_{m+1}\}$ // construct new set of speciation rates
$\mu' = \{\mu \cup \mu_{m+1}\}$ // construct new set of extinction rates
$\mathbf{s}' = \{\mathbf{s} \cup s_{m+1}\}$ // construct new set of shift events
$f(\lambda', \mu', \mathbf{s}') \propto \text{Likelihood}(\lambda', \mu', \mathbf{s}') \times \text{Prior}(\lambda', \mu', \mathbf{s}')$ // compute the posterior probability for the proposed state
$f(\lambda, \mu, \mathbf{s}) \propto \text{Likelihood}(\lambda, \mu, \mathbf{s}) \times \text{Prior}(\lambda, \mu, \mathbf{s})$ // compute the posterior probability for the current state
$q(\lambda', \mu', \mathbf{s}' | \lambda, \mu, \mathbf{s}) = 1/t_i \times \text{Prior}(\lambda_{m+1}, \mu_{m+1}) \times 1/(2N-1) \times \beta$ // compute the forward proposal probability where $\beta = 1$ if $m = 0$ and $\beta = 0.5$ otherwise
$q(\lambda, \mu, \mathbf{s} | \lambda', \mu', \mathbf{s}') = 1/(m+1)$ // compute the reverse proposal probability
$\text{J} = 1$ // compute the Jacobian
$\mathbf{R} = \frac{f(\lambda', \mu', \mathbf{s}')}{f(\lambda, \mu, \mathbf{s})} \times \frac{q(\lambda, \mu, \mathbf{s} | \lambda', \mu', \mathbf{s}')}{q(\lambda', \mu', \mathbf{s}' | \lambda, \mu, \mathbf{s})} \times J$
$\quad = \frac{f(\lambda', \mu', \mathbf{s}')}{f(\lambda, \mu, \mathbf{s})} \times \frac{1}{(m+1) \times 1/t_i \times \text{Prior}(\lambda_{m+1}, \mu_{m+1}) \times 1/(2N-1) \times \beta}$ // compute the acceptance probability where $\beta = 0.5$ if $m = 0$ and $\beta = 1$ otherwise

Algorithm S2 Death move

Inputs:
m: the current number of diversification-rate shifts.

$i \sim \text{unif}(1, m)$ // randomly select one of the m current diversification-rate shifts to delete
$\lambda' = \{\lambda \backslash \lambda_i\}$ //
$\mu' = \{\mu \backslash \mu_i\}$ //
$\mathbf{s}' = \{\mathbf{s} \backslash s_i\}$ //
$\lambda' = \{\lambda \cup \lambda_{m+1}\}$ // construct new set of speciation rates
$\mu' = \{\mu \cup \mu_{m+1}\}$ // construct new set of extinction rates
$\mathbf{s}' = \{\mathbf{s} \cup s_{m+1}\}$ // construct new set of shift events
$f(\lambda', \mu', \mathbf{s}') \propto \text{Likelihood}(\lambda', \mu', \mathbf{s}') \times \text{Prior}(\lambda', \mu', \mathbf{s}')$ // compute the posterior probability for the proposed state
$f(\lambda, \mu, \mathbf{s}) \propto \text{Likelihood}(\lambda, \mu, \mathbf{s}) \times \text{Prior}(\lambda, \mu, \mathbf{s})$ // compute the posterior probability for the current state
$q(\lambda', \mu', \mathbf{s}' | \lambda, \mu, \mathbf{s}) = 1/m$ // compute the forward proposal probability
$q(\lambda, \mu, \mathbf{s} | \lambda', \mu', \mathbf{s}') = 1/t_i \times \text{Prior}(\lambda_i, \mu_i) \times 1/(2N-1) \times \beta$ // compute the reverse proposal probability where $\beta = 1$ if $m = 1$ and $\beta = 0.5$ otherwise
$\text{J} = 1$ // compute the Jacobian
$\mathbf{R} = \frac{f(\lambda', \mu', \mathbf{s}')}{f(\lambda, \mu, \mathbf{s})} \times \frac{q(\lambda, \mu, \mathbf{s} | \lambda', \mu', \mathbf{s}')}{q(\lambda', \mu', \mathbf{s}' | \lambda, \mu, \mathbf{s})} \times J$
$\quad = \frac{f(\lambda', \mu', \mathbf{s}')}{f(\lambda, \mu, \mathbf{s})} \times (m+1) \times \frac{1}{t_i} \times \text{Prior}(\lambda_{m+1}, \mu_{m+1}) \times 1/(2N-1) \times \beta$ // compute the acceptance probability where $\beta = 0.5$ if $m = 0$ and $\beta = 1$ otherwise

S2 BDS Model Parameterization

The birth-death-shift (BDS) process includes three types of events (speciation, extinction, and rate-shifts) that are governed by their respective rates. The speciation and extinction rates are drawn from some base distribution, whereas the shift-rate is constant over the entire phylogeny. Our implementation in `RevBayes` [2] uses K rate categories to approximate the continuous base distribution. In principle, any set of K rates can be used. In Listing 1 we provide the full model specification. In sections below we detail each model choice and discuss alternatives.

The implementation of our methods in `RevBayes` affords users considerable flexibility in applying specifying different BDS models that specify what processes (speciation and/or extinction, or net-diversification rates) change during a diversification-rate shift. We expect that the choice of a specific BDS model will be made by comparing the relative fit of candidate models to the dataset at hand (e.g., using Bayes factors).

```
# Global parameters for our analysis
K = 10            # number of rate categories
H = 0.587405

# speciation and extinction rates
speciation_mean ~ dnUniform( 0, 1E2)
extinction_mean ~ dnUniform( 0, 1E2)
speciation_rate_sd ~ dnExponential( 1.0 / H )
extinction_rate_sd ~ dnExponential( 1.0 / H )
speciation_cats := fnDiscretizeDistribution(
                       dnLognormal(ln(speciation_mean), speciation_rate_sd),
                       K )
extinction_cats := fnDiscretizeDistribution(
                       dnLognormal(ln(extinction_mean), extinction_rate_sd),
                       K )

index = 1
for (i in 1:K) {
    for (j in 1:K) {
        speciation[index] := speciation_cats[i]
        extinction[index] := extinction_cats[j]
        index += 1
    }
}

### Create uniform probabilities for the category at the root
rate_cat_probs <- simplex( rep(1, K*K) )

### Set the rate of rate-shift events to 0.0
shift_rate ~ dnUniform(0.0, 100.0/TL)

### construct a variable for the tree drawn from a birth-death-shift process
timetree ~ dnBDS( rootAge = root,
        speciationRates   = speciation,
        extinctionRates   = extinction,
        Q                 = fnJC(K*K),
        delta             = shift_rate,
        pi                = rate_cat_probs,
        condition         = "time")
```

Listing 1: Full specification of the BDS process with lognormal distributed speciation and extinction rates.

In line 6, we define a log-uniform hyperprior distribution on the prior mean of the speciation-rate distribution, and in line 7 we use the same hyperprior on the prior mean of the extinction-rate distribution. Next, in line 8 and 9, we specify an exponential hyperprior distribution on the standard deviation of the speciation-rate prior distribution and extinction-rate prior distribution, respectively. Now we have specified parameters of the base distributions for the speciation and extinction rates. Any other hyperprior distribution can be applied here, or these parameters might also be fixed to constant values (see Section S2.1.2).

We continue in lines 10–15 to discretize the base distribution for the speciation and extinction rates using the function `fnDiscretizeDistribution`. This function, `fnDiscretizeDistribution`, can be applied to any distribution with a known quantile function (see Section S2.1.3). In this case, we chose a lognormal distribution as the base distribution for the speciation and extinction rates. Additionally, we provide the number of discrete categories to be used in discretizing the base distribution. Now we have the K (here we used $K = 10$) speciation and extinction rates computed as the quantiles of lognormal distribution.

The next step is to set up the pairs of speciation and extinction rates for each rate category. Since we want to allow that speciation and extinction rates change independently, we need to build $K \times K$ pairs. Thus, we actually use K^2 rate categories in our computation because for each speciation rate λ_k we find K possible extinction rates μ_j. We set up these paired rate categories in lines 17–24.

Next, we specify equal probabilities as the prior probability for each rate category at the root (line 27). We discuss alternatives in Section S2.2. Furthermore, we specify a uniform prior on the shift rate η (line 30). This prior distribution implies that under a Poisson process we would expect between 0 and 100 shifts along the phylogeny. Note that the birth-death-shift process does not follow exactly a Poisson distribution and this is rather an approximation. We discuss alternatives in Section S2.3.

Finally, we combine all of the parameters to set-up the birth-death-shift process (lines 33–39). We additionally require the age of the root which is used for initialization and simulation under the BDS process. Since we treat the tree as an observation, we can simply use the "observed" root age. This feature is of relevance when we want to use the BDS process as a tree-prior for divergence time estimation. The next new parameters is the rate matrix Q (line 36). For now we simply use equal rates to switch from any rate category i to j. Equal switch rates between rates categories follow from the assumption that rates are drawn from a continuous base distribution. If the biologist wanted to use K rate categories that simply represent distinct rate categories but not approximate a base distribution, then it will be of interest to allow for non-equal switch rates.

In the last line we condition the process. Here, we condition on time, which is equivalent to not conditioning on anything (other than the condition that process has evolved for a certain amount of time). In that case, the full probability of the tree under the BDS process is computed as

$$f(\Psi) = \sum_{j=1}^{K} D_{2N-1,j}(t_{2N-1})\pi_j. \tag{S2}$$

We also provide the possibility to condition on survival of the process; that is, we condition on non-extinction by dividing by the marginal probability that both descendants of the root node leave at least one descendant:

$$f(\Psi|N(t_1) > 0) = \frac{\sum_{j=1}^{K} D_{2N-1,j}(t_{2N-1})\pi_j}{1 - \sum_{j=1}^{K} E^2_{2N-1,j}(t_{2N-1})\pi_j} \tag{S3}$$

S2.1 Alternative parameterizations of the speciation- and extinction-rate categories

S2.1.1 Constant speciation and extinction rates

We show in Listing 2 how to specify constant speciation and extinction rates. That is, we simply specify "replicate" the same speciation and extinction rate K times using the function `rep`. This example is identical to specifying a constant-rate birth-death process, which we use to validate our implementation. Furthermore, the example can be used if one wants to set either the speciation or extinction rate to be constant but let the other rate vary.

```
# Global parameters for our analysis
N = 367          # total number of species
K = 10           # number of rate categories
H = 0.587405

# speciation and extinction rates
speciation_mean <- 2.0 * (N-2) / TL
extinction_mean <- (N-2) / TL
speciation := rep(speciation_mean, K)
extinction := rep(extinction_mean, K)
```

Listing 2: BDS process specified with constant speciation and extinction rates.

S2.1.2 Lognormally distributed speciation and extinction rates with fixed hyperparameters

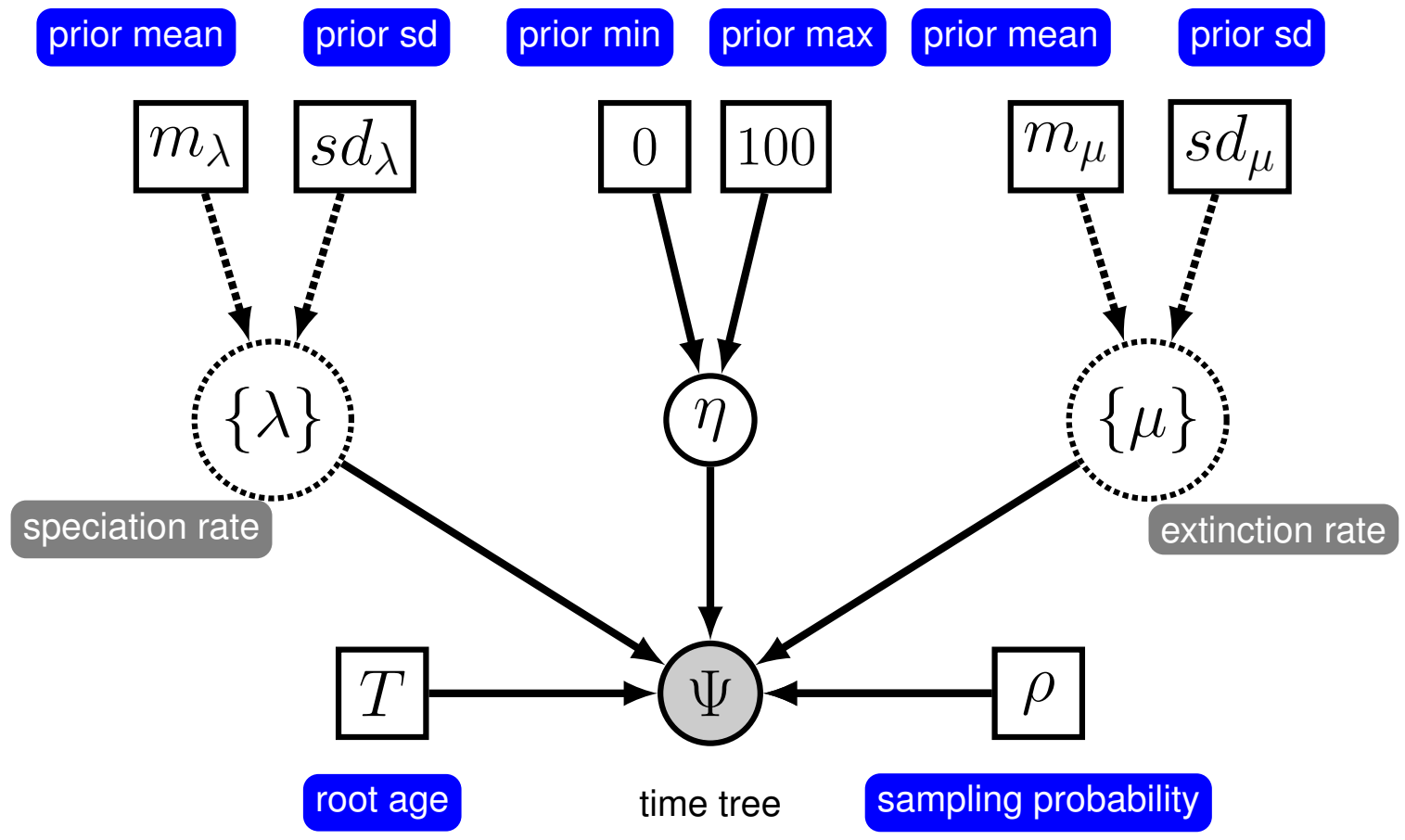

Figure S1: A graphical model representing the birth-death-shift model with fixed hyperparameters. The vector of branch-specific speciation rates $\{\lambda\}$ are drawn from a lognormal distribution with parameters m_λ and sd_λ. Similarly, the branch-specific extinction rates are drawn from a lognormal distribution with parameters m_μ and sd_μ. We show $\{\lambda\}$ and $\{\mu\}$ as deterministic nodes because they represent k values from a discretized lognormal distribution. Note that here the hyperparameters are fixed, thus, the only free parameter in the model for the likelihood computation is the shift rate η. Although the prior distribution and thus the vector of speciation and extinction rates is fixed (similar to `BAMM`), the actual rates on the branches are mapped/estimated using the stochastic-mapping algorithm.

In the main example, we used a hyperprior distribution on the mean and standard deviation of the lognormal base distribution. However, we could also fix the mean and standard deviation of the base distributions. For example, we could set the mean speciation rate to twice the maximum likelihood estimate under a pure-birth process, $2(N-2)/TL$.

```
# speciation and extinction rates
H = 0.587405
speciation_mean <- 2.0 * (N-2) / TL
extinction_mean <- (N-2) / TL
speciation := fnDiscretizeDistribution(
                        dnLognormal(ln(speciation_mean), H),
                        K )
extinction := fnDiscretizeDistribution(
                        dnLognormal(ln(extinction_mean), H),
                        K )
```

Listing 3: BDS process specified with lognormal distributed speciation and extinction rates with a fixed mean and variance.

S2.1.3 Alternative base distributions for speciation and extinction rates

We chose to model speciation and extinction rates under a lognormal distribution. The lognormal distribution has several advantages: it is only defined for positive, real numbers, and has a separate parameter to specify the mean and variance. Otherwise, there is no consensus in the literature regarding the propriety of various prior distributions for diversification rates. The only strict requirement is that the distribution can only be defined for positive real numbers (because negative diversification rates are meaningless). In `RevBayes` we provide the following options: (1) Half-Cauchy (2) Half-Normal (3) χ^2 (4) Exponential (5) Gamma (6) Inverse-Gamma (7) Log-Normal (8) Log-Uniform (9) StudentT (10) Truncated-Normal (11) Uniform.

As a demonstration, we show in Listing 4 how to use an exponential base distribution with fixed mean parameters as the base distribution. The exponential base distribution with fixed mean parameter is the currently implemented option in `BAMM` [3]. See Kopperud and Höhna [4] for an exploration of different base distributions.

```
# speciation and extinction rates
speciation_mean <- 2.0 * (N-2) / TL
extinction_mean <- (N-2) / TL
speciation := fnDiscretizeDistribution(
                        dnExponential( 1.0/speciation_mean ),
                        K )
extinction := fnDiscretizeDistribution(
                        dnExponential( 1.0/extinction_mean),
                        K )
```

Listing 4: BDS process specified with exponential distributed speciation and extinction rates with a fixed mean.

S2.1.4 Dependent rate categories

Finally, we could parameterize the BDS model where speciation and extinction rates are assumed to vary dependently across the tree. Under this model, a diversification-rate shift involves a change from one pair of rates (λ_i, μ_i), (where i corresponds to the same discrete rate category of both base distributions) to a new pair of speciation and extinction rates (λ_j, μ_j). For example, a diversification-rate shift might involve a change from paired rates (λ_3, μ_3) to (λ_5, μ_5) (reflecting a shift from the third to the fifth discrete categories of the speciation- and extinction-rate base-probability distributions).

```
# speciation and extinction rates
H = 0.587405
speciation_mean ~ dnUniform( 0, 1E2)
extinction_mean ~ dnUniform( 0, 1E2)
speciation_rate_sd ~ dnExponential( 1.0 / H )
extinction_rate_sd ~ dnExponential( 1.0 / H )
speciation := fnDiscretizeDistribution(
                        dnLognormal(ln(speciation_mean), speciation_rate_sd),
                        K )
extinction := fnDiscretizeDistribution(
                        dnLognormal(ln(extinction_mean), extinction_rate_sd),
                        K )
```

Listing 5: BDS process specified with lognormal distributed speciation and extinction rates with a fixed mean and variance.

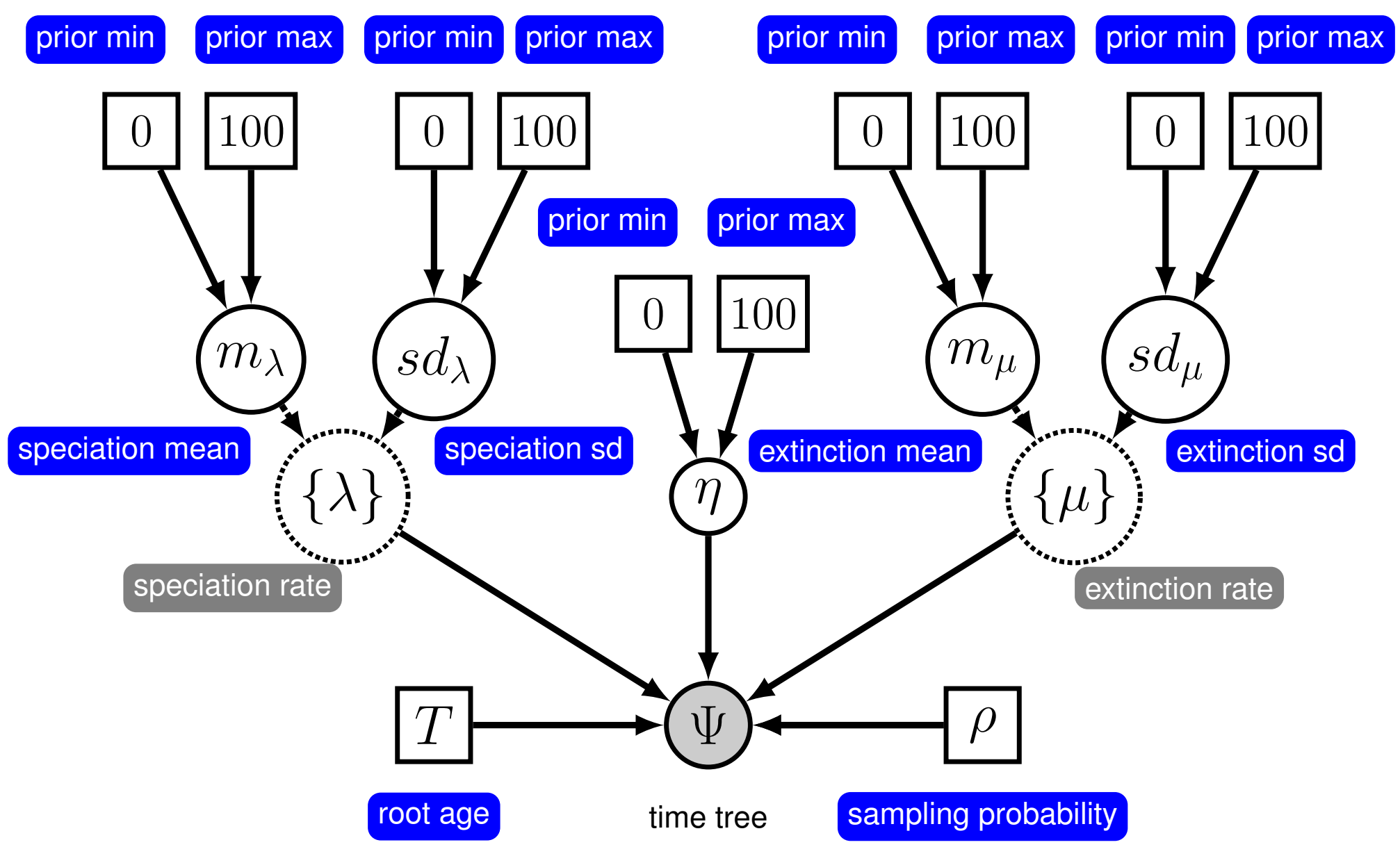

Figure S2: A graphical model representing the birth-death-shift model with dependent rate categories. The vector of branch-specific speciation rates $\{\lambda\}$ are drawn from a lognormal distribution with parameters m_λ and sd_λ. Similarly, the branch-specific extinction rates are drawn from a lognormal distribution with parameters m_μ and sd_μ. We show $\{\lambda\}$ and $\{\mu\}$ as deterministic nodes because they represent k values from a discretized lognormal distribution. We arbitrarily choose a uniform hyperprior distribution for the hyperparameters m_λ, sd_λ, m_μ and sd_μ. The shift rate η is also drawn from a uniform distribution. The important aspect of this model is the pairing of speciation and extinction rates; i.e., a rate shift goes from low to high rate categories for both speciation and extinction rates.

S2.2 Root prior

In our examples, we used an equal prior probability on the diversification-rate category at the root. However, we implemented the root probability as a variable that the user can either fix to a preferred value, or estimate by specifying a prior distribution on it. We refer the reader to the discussion in the literature on specifying root categories under state-dependent speciation and extinction processes [5, 6, 7, 8, 9, 10].

```
### Create equal probabilities for the category at the root
rate_cat_probs <- simplex( rep(1, K) )

#### Alternatively, we could estimate the root probability
rate_cat_probs ~ dnDirichlet( rep(1, K) )
```

Listing 6: Different options to specify the root category probabilities.

S2.3 Shift Rate

Finally, we provide different options and recommendations for specifying the shift rate. The shift rate ν is parameter defines the rate of events in the BDS process. Again, in `RevBayes` this rate parameter can either be fixed or estimated. In some of our examples, we either set the shift rate to zero, $\nu = 0.0$, to disallow any rate shifts (and thus simplifies the BDS process to a constant-rate birth-death process). In other examples, we provided a fixed rate so that the expected number of events under a Poisson process would be, for example, two, $\nu = 2.0/TL$. Lastly, we can specify any type of prior distribution on the shift rate; for example, we can specify a uniform prior distribution so that the expected number of events varies between 0 and 100, $\nu \sim \text{dnUniform}(0.0, 100.0/TL)$.

```

### Set the rate of rate-shift events to 0.0
shift_rate <- 0.0

### Set the rate of rate-shift events to one expected event
shift_rate <- 1.0 / TL

### Set the rate of rate-shift events to two expected events
shift_rate <- 2.0 / TL

### Set a variable rate of rate-shift events
### between 0 and 100 expected events across the phylogeny
shift_rate ~ dnUniform(0.0, 100.0/TL)
```

Listing 7: Different options to specify the shift rate.

S3 Validating the BDS Method

S3.1 Validating with special cases of the BDS process

3.1.1 Special-case validation of BDS likelihoods

Goal & Rationale—The foundation of any likelihood-based (i.e., maximum likelihood or Bayesian) method is the ability to correctly compute the probability of observing the dataset at hand under the inference model. It is therefore critical to validate the theory and implementation of our likelihood calculations under the BDS process. Ideally, we would validate our BDS likelihood calculations by comparing them to analytical results. Unfortunately, analytical results are not available for the BDS process (which, of course, is why we developed numerical methods to calculate likelihoods in the first place). Analytical results are, however, available for the constant-rate birth-death process [11, 12]. It is possible to restrict the BDS process to a constant-rate birth-death process, which would provide a "special-case" analytical benchmark for our likelihood calculations. Specifically, the BDS process simplifies to a constant-rate birth-death process when the event rate is set to zero ($\eta = 0$) and the diversification rates are identical for each rate category ($\lambda_i = \lambda_j$ and $\mu_i = \mu_j$ for all i, j). (Note that the latter restriction is necessary to specify the constant-rate birth-death process, as we would otherwise obtain a mixture of constant-rate birth-death processes.) We performed this special-case validation experiment (as described below).

Data—We performed our experiments on the dated phylogeny of primates published by Springer et al. [13], where we treated this phylogeny as an observation; i.e., the data used to compute analytical likelihoods under the constant-rate birth-death process and to estimate likelihoods under our restricted BDS process.

Inference model—The restricted BDS model for this validation experiment assumes that there are no rate shifts, so we set the event-rate parameter, $\eta = 0$. To specify the other parameters of the restricted BDS process—the speciation and extinction rates—we used an empirical-hyperprior approach. That is, we first calculated the maximum-likelihood estimate for the speciation rate from the primate tree under a constant-rate birth-death process with assumed complete taxon sampling. Specifically, the MLE for the speciation rate, $\hat{\lambda}$, under a constant-rate birth-death process with complete taxon sampling is calculated as $\hat{\lambda} = (N-2)/TL$, where N is the number of species (367) and TL the tree length (sum of branch durations) in the dated primate tree. We then fixed the speciation rate for all rate categories to be equal to twice the MLE of the speciation rate, $\lambda_i = 2 \times \hat{\lambda}$. Next, we specified a range of values for the extinction rate, μ_i, with 1000 incremental values ranging from 0.0 to λ_i; i.e., $\mu = \{0.000, 0.001 \ldots 1.000\} \times \lambda_i$. We then estimated likelihoods for the primate tree under 1000 unique parameterizations of our restricted BDS process; i.e., corresponding to each unique value of the relative-extinction rate $\epsilon = \mu_i \div \lambda_i$. The BDS model parameters are listed in Table S1.

Table S1: Parameter names and values for the restricted BDS model.

Parameter	X	Value
Speciation rates	λ_i	$2 \times \hat{\lambda}$
Extinction rates	μ_i	$\{0.000, 0.001, 0.002, \ldots, 0.999, 1.000\} \times \lambda_i$
Shift rate	η	0.0

Analyses—We performed two sets of analyses: we first estimated likelihoods using our numerical-integration algorithm (which uses the post-order ODE algorithm to to average over all possible diversification-histories), and in the second set of analyses we estimated likelihoods using our data-augmentation algorithm (which uses rjMCMC to average over all possible diversification-histories). For these analyses, we assumed $K = 4$ discrete rate categories and arbitrarily set the root category to 1 (note that this arbitrary choice for the root category is inconsequential because the diversification rates for each category were restricted to be equal). We provide the scripts necessary to perform these analyses in the `1_likelihood` directory of our GitHub repository: https://github.com/hoehna/birth-death-shift-analyses.

Results—The analytical likelihoods under the constant-rate birth-death process are identical to the likelihoods that we estimated under the restricted BDS process using both our numerical-integration and data-augmentation algorithms (Figure S3).

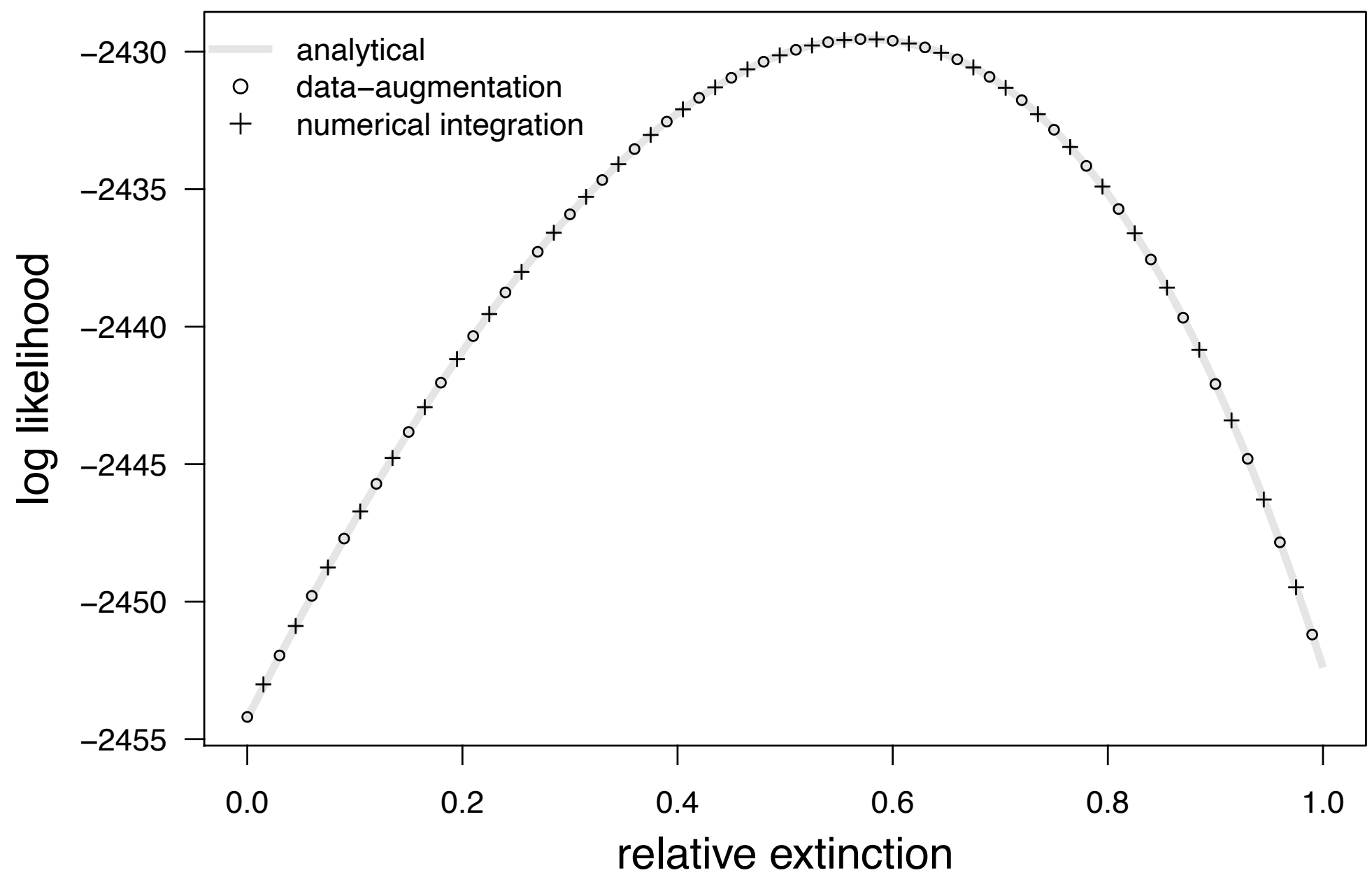

Figure S3: Special-case validation of BDS likelihoods We computed analytical likelihoods of the primate tree under a constant-rate birth-death process over a range of values for the the relative-extinction rate, $\epsilon = \mu \div \lambda$ (shaded line). We estimated the same set of likelihoods under a restricted BDS model where the event rate is zero ($\eta = 0$) and all K rate categories have identical rates ($\lambda_i = \lambda_j$ and $\mu_i = \mu_j$). We estimated these BDS likelihoods using both the numerical-integration method (+ symbols) and the data-augmentation method (∘ symbols). The analytical and estimated likelihoods are identical, validating both the theory and implementation of the BDS likelihood calculations.

3.1.2 Special-case validation of the number of diversification-rate shifts

Goal & Rationale—We performed a second experiment to confirm that the BDS process correctly models diversification-rate shifts. Ideally, we would perform this validation by means of an analytical benchmark. We might expect that the Poisson distribution provides an appropriate analytical benchmark. That is, perhaps the distribution for the number of diversification-rate shifts, S, over the entire tree follows a Poisson distribution with rate $\Lambda = \eta TL$. Although the Poisson distribution correctly describes the distribution of events that are conditionally independent of the tree (e.g., such as when we simulate trait evolution over the tree), it will not correctly describe the distribution of events that change the tree-generating process [14]. This is because a rate-shift event will impact the tree length, e.g., a shift to a higher speciation rate will, most likely, increase the tree length. However, we could use this analytical benchmark to validate the number of diversification-rate shifts under a (different) restricted BDS process; i.e., one that allows rate shifts ($\eta \neq 0$), but specifies equal diversification rates for each category ($\lambda_i = \lambda_j$ and $\mu_i = \mu_j$). When diversification rates are the same for each rate category, the inferred posterior distribution for the number of "quasi" rate shifts should be identical to its analytical Poisson distribution. We performed this special-case validation experiment (as described below).

Data—We again performed our experiments on the dated phylogeny of primates published by Springer et al. [13], where we treated this phylogeny as an observation; i.e., the data used to estimate the number of diversification-rate shifts under our restricted BDS process.

Inference model—The restricted BDS model for this validation experiment allows quasi rate shifts among a set of identical diversification-rate categories. We specified three values for the expected number of diversification-rate shifts over the tree, $E(S) = \{1, 10, 20\}$, where the corresponding event rate is $\eta = E(S)/TL$. As before, we first calculated the MLE for the speciation rate from the primate tree under a constant-rate birth-death process with complete taxon sampling: $\hat{\lambda} = (N-2)/TL$, where N is the number of species (367) and TL the tree length. We then fixed the speciation rate for all rate categories to be equal to twice the maximum likelihood estimate of the speciation rate, $\lambda_i = 2 \times \hat{\lambda}$. Finally, we fixed the extinction rate to be half the speciation rate, $\mu_i = \lambda_i/2$. The parameters for this restricted BDS model are listed in Table S2.

Table S2: Parameter names and values for the restricted BDS model.

Parameter	X	Value
Speciation rate	λ_i	$2 \times \hat{\lambda}$
Extinction rate	μ_i	$\lambda_i/2$
Shift rate	η	$E(S)/TL^1$

[1] TL is the tree length (i.e., sum of the branch durations) of the primate phylogeny.

Analyses—We performed six sets of analyses; one for each expected number of diversification-rate shifts, $E(S) = \{1, 10, 20\}$ by the two BDS algorithms (the stochastic-mapping and data-augmentation methods). For each unique analysis (i.e., combination of $E(S)$ and inference method), we estimated the posterior distribution of the number of diversification-rate shifts by performing 8 replicate MCMC simulations. For these analyses, we assumed $K = 4$ rate categories and arbitrarily set the root category to 1 (this arbitrary choice is inconsequential because the diversification rates for each category were restricted to be equal). We provide the scripts necessary to perform these analyses (with details of the proposal mechanisms, chain length, etc.) in the `2_shift_prior` directory of our GitHub repository: https://github.com/hoehna/birth-death-shift-analyses.

Results—Our experiments confirm that the inferred distributions of the number of rate shifts under this restricted BDS process matches the corresponding analytical Poisson distributions (Figure S4).

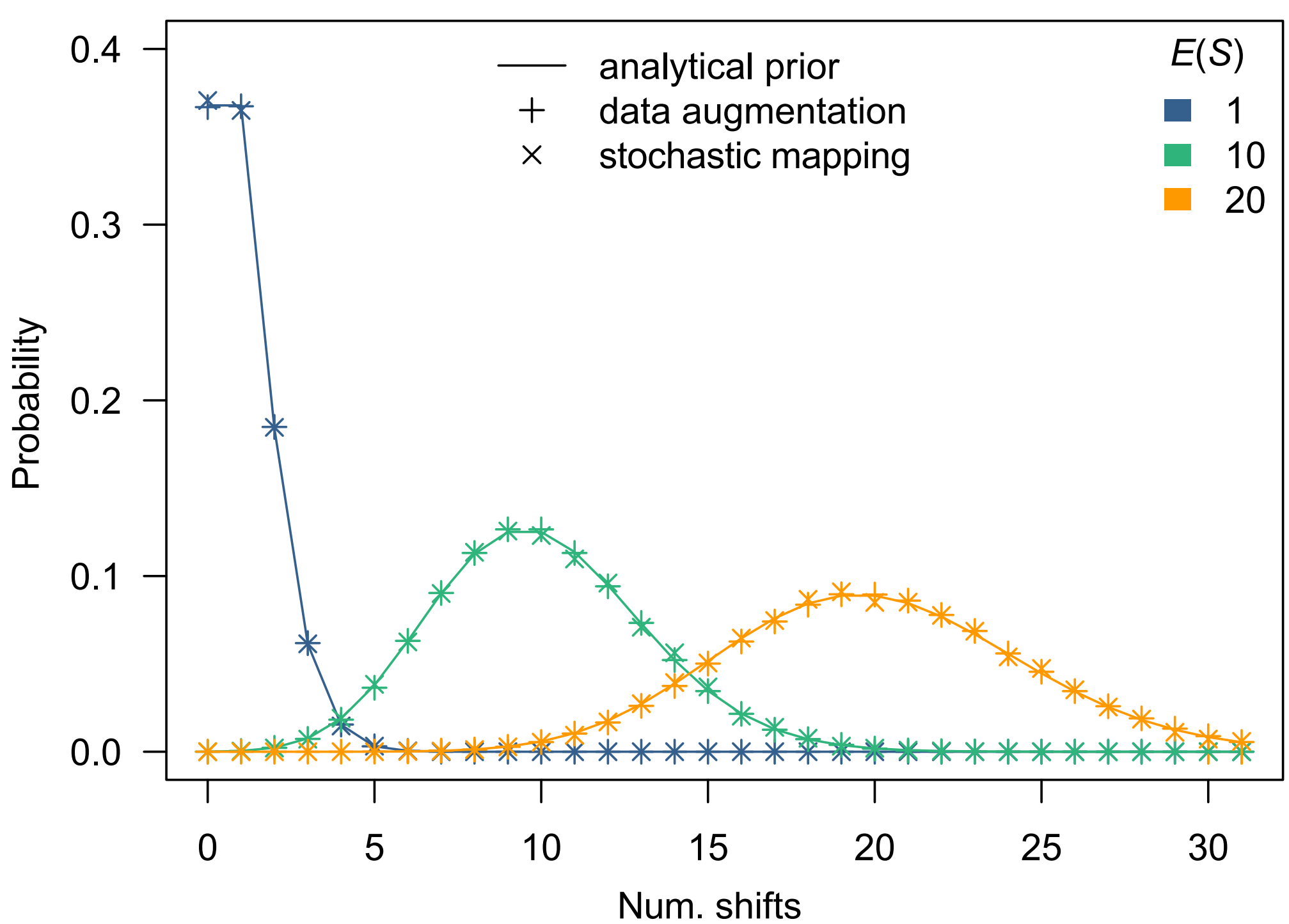

Figure S4: Special-case validation of the number of diversification-rate shifts. We used an analytical benchmark—the Poisson distribution—to validate the distribution of diversification-rate shifts under a restricted BDS process; this restricted BDS process allows rate shifts ($\eta \neq 0$), but specifies equal diversification rates for each category ($\lambda_i = \lambda_j$ and $\mu_i = \mu_j$). We estimated the number of diversification-rate shifts under this restricted BDS model using both the stochastic-mapping method ($\times$ symbols) and the data-augmentation method ($+$ symbols) with the prior on η specified so that the expected number of diversification-rate shifts were $E(S) = \{1, 10, 20\}$. The estimated distributions are identical to the corresponding analytical distributions, confirming the correctness of the derivation and implementation of the independent BDS methods.

S3.2 Validating with a second algorithm

Goal & Rationale—The preceding experiments provide compelling evidence that the theoretical derivation and implementation of our BDS process are correct, given certain special-case restrictions; e.g., where $\eta = 0$ and/or equal rates ($\lambda_i = \lambda_j$ and $\mu_i = \mu_j$) for all K rate categories. Our goal here is to validate the derivation and implementation of the likelihood function for the *unrestricted* BDS process (where diversification rates are free to vary among lineages). To this end, we developed a second, mathematically equivalent approach for calculating the likelihood of an observed tree under the BDS process. Specifically, this 'data-augmentation' algorithm calculates the probability of an observed tree by averaging over all possible diversification histories under the BDS process. The details of our data-augmentation algorithm are detailed in Section S1 of this document. We can use this independent, mathematically equivalent method to validate our stochastic-mapping algorithm: i.e., because the data-augmentation and stochastic-mapping approaches are mathematically equivalent, branch-specific diversification rates inferred using these two independent methods—if they are correct—should be identical. We performed this validation experiment (as described below).

Data—We performed these validation experiments on the dated phylogeny of primates published by Springer et al. [13], where we treated this phylogeny as an observation; i.e., the data for estimating branch-specific speciation rates under the BDS process using both the data-augmentation and stochastic-mapping approaches.

Inference model—Our estimates of branch-specific diversification rates in the primate tree—using both the data-augmentation and stochastic-mapping approaches—are based on an identically specified BDS inference model. Specifically, we specified the event-rate parameter, $\eta = 2/TL$, such that the expected number of diversification-rate shift is $E(S) = 2$. We specified a lognormal distribution for the speciation rate with a fixed mean $\theta_\lambda = \hat{\lambda}$ (where $\hat{\lambda} = (N-2)/TL$, the MLE of the speciation rate estimated from the primate tree), and a fixed standard deviation $\sigma_\lambda = 0.58740$ (such that the 95% prior interval spans one order of magnitude [15]). We discretized the lognormal distribution for the speciation rate using K equiprobable rate categories (i.e., quantiles). For our analyses, we explored a range of discrete categories; $K = \{4, 6, 8, 10, 12, 20\}$. For a given number of rate categories, K, we specified the value of the i-th speciation-rate category as the median of each quantile; $p = i/K - 0.5/K$, and fixed the extinction rate to be half that of the mean speciation rate, $\mu_i = \hat{\lambda}/2$. We used a uniform prior on the rate category at the root, Unif$(1, K)$. We list the BDS model parameters in Table S3.

Table S3: Parameter names and priors/values for the BDS model.

Parameter	X	$f(X)$
Speciation rates	λ_i	qlnorm$(p, \hat{\lambda}, 0.587405)$
Extinction rates	μ_i	$\hat{\lambda}/2.0$
Shift rate	η	$2/TL$[1]
Root category	r	Unif$(1, K)$

[1] TL is the length of the primate tree.

Analyses—For each value of K, we performed two sets of analyses to estimate the branch-specific speciation rates in the primate tree: one set of analyses used the the data-augmentation algorithm, and the other used the stochastic-mapping algorithm. For each unique combination of K and algorithm, we estimated the joint posterior probability distribution using 32 replicate MCMC simulations to achieve higher precision in our estimates. We provide the scripts necessary to perform these analyses (with details of the proposal mechanisms, chain length, etc.) in the `3_branch_rates` directory of our GitHub repository: https://github.com/hoehna/birth-death-shift-analyses.

Results—Our analyses using the two independent algorithms inferred identical branch-specific diversification rates in the primate phylogeny (Figure S5).

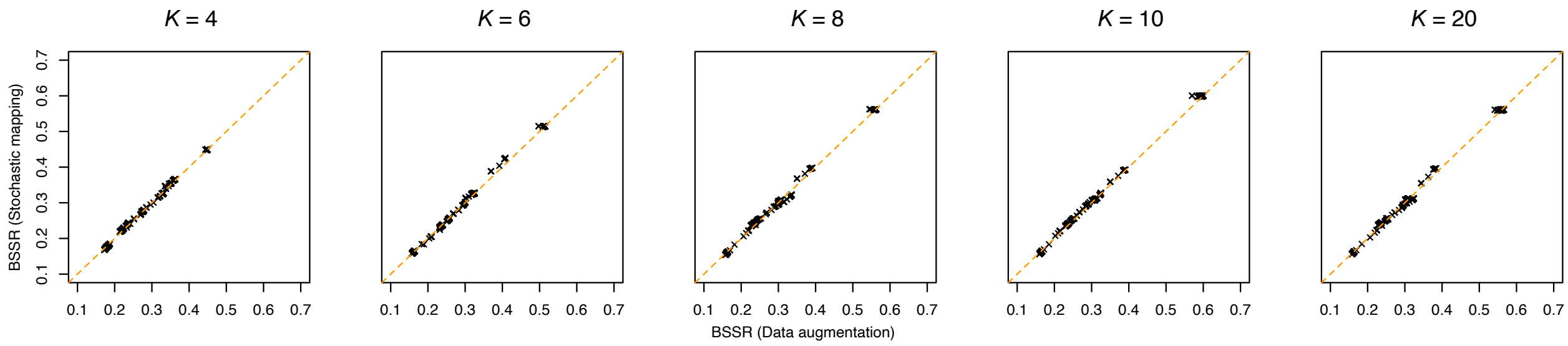

Figure S5: Validating the theory and implementation of our BDS process with a second algorithm. We estimated branch-specific speciation rates (BSSR) in the primate tree using both our stochastic-mapping algorithm and a second, mathematically equivalent data-augmentation algorithm. Each plot depicts results for a specific number of discrete rate categories, $K = \{4, 6, 8, 10, 20\}$. Each point indicates the average speciation rate for a single branch estimated using the data-augmentation algorithm (x-axes) against the corresponding estimate using the stochastic-mapping algorithm (y-axes); the dashed line indicates perfect agreement. Both methods provide identical branch-specific speciation-rate estimates.

Comparing the Computational Efficiency of the Two Numerical Methods

Goal & Rationale—Given that we have developed two approaches for estimating branch-specific diversification rates under the BDS process—based on the stochastic-mapping and data-augmentation algorithms—we wanted to assess their relative computational efficiency. We performed experiments to explore this issue (as described below).

Data—We performed analyses of six empirical phylogenies using both the stochastic-mapping and data-augmentation algorithms. We list these empirical datasets in Table S4.

Table S4: Datasets used to explore relative computational efficiency

Dataset	Study Group	Num. Species	Study
1	*Byttneria*	35	[16]
2	Cetaceans	87	[17]
3	*Viburnum*	117	[18]
4	Primates	367	[13]
5	Ericaceae	450	[19]
6	Conifers	492	[20]

Inference model—We explored the relative computational efficiency of the data-augmentation and stochastic-mapping approaches by performing analyses of the six empirical datasets under identically specified BDS inference models. Specifically, we specified three values for the expected number of diversification-rate shifts, $E(S) = \{1, 10, 20\}$, where the corresponding event-rate parameter is $\eta = E(S)/TL$. We specified a lognormal distribution for the speciation rate with a fixed mean $\theta_\lambda = \hat{\lambda}$ (where $\hat{\lambda}$, is the MLE of the speciation rate), and a fixed standard deviation $\sigma_\lambda = 0.58740$ (such that the 95% prior interval spans one order of magnitude [15]). We discretized the lognormal distribution for the speciation rate using K equiprobable categories (i.e., quantiles). For our analyses, we explored a range of discrete categories; $K = \{2, 4, 6, 8, 10\}$. For a given number of rate categories, K, we specified the value of the i-th speciation-rate category as the median of each quantile; $p = i/K - 0.5/K$, and fixed the extinction rate to be half that of the mean speciation rate, $\mu_i = \hat{\lambda}/2$. We used a uniform prior on the rate category at the root, Unif$(1, K)$. We list the BDS model parameters in Table S5.

Analyses—For each of the six datasets, we performed 30 sets of analyses; i.e., for all combinations of the five rate categories, $K = \{2, 4, 6, 8, 10\}$, by all three values for the expected number of rate shifts, $E(S) = \{1, 2, 10\}$, by the two inference methods (the data-augmentation and stochastic-mapping algorithms). For each unique combination of K, $E(S)$, and algorithm, we estimated the joint posterior probability distribution using 32 replicate MCMC simulations. We provide the scripts necessary to perform these analyses (with details of the proposal mechanisms, chain length, etc.) in the `4_ESS` directory of our GitHub repository: https://github.com/hoehna/birth-death-shift-analyses.

Table S5: **Parameter names and priors/values for the BDS model.**

Parameter	X	$f(X)$
Speciation rates	λ_i	qlnorm$(p, \hat{\lambda}, 0.587405)$
Extinction rates	μ_i	$\hat{\lambda}/2.0$
Shift rate	η	$\{1/TL, 2/TL, 10/TL\}^1$
Root category	r	Unif$(1, K)$

[1]TL is the length of the primate tree.

Results—To compare the relative computational efficiency of the two methods, we used a summary statistic based on the ratio of the average ESS values for the data-augmentation and stochastic-mapping algorithms. Specifically, for each method—the data-augmentation or stochastic-mapping algorithm—we first combine all of the samples from the 32 replicate MCMC simulations for a given unique analysis (i.e., unique combination of $E(S)$ and K). We then compute the Effective Sample Size (ESS) for every parameter, which is calculated as the number of MCMC samples for that parameter divided by the Autocorrelation Time for that parameter. Next, we calculate the average of the ESS values for all parameters for the model used in that unique analysis. We will now have computed—for both algorithms—the average ESS value for a specific analysis, where the average is over all of the BDS parameters for that analysis. Finally, for each unique analysis, we compute a summary statistic that is the ratio of the two corresponding average ESS values: $\overline{ESS}_{SM} \div \overline{ESS}_{DA}$. We summarize these results in Figure S6. Our experiments indicate that the stochastic-mapping method is $\approx 20 - 500$ times more computationally efficient than the data-augmentation method, where the relative computational advantage appears to scale with tree size.

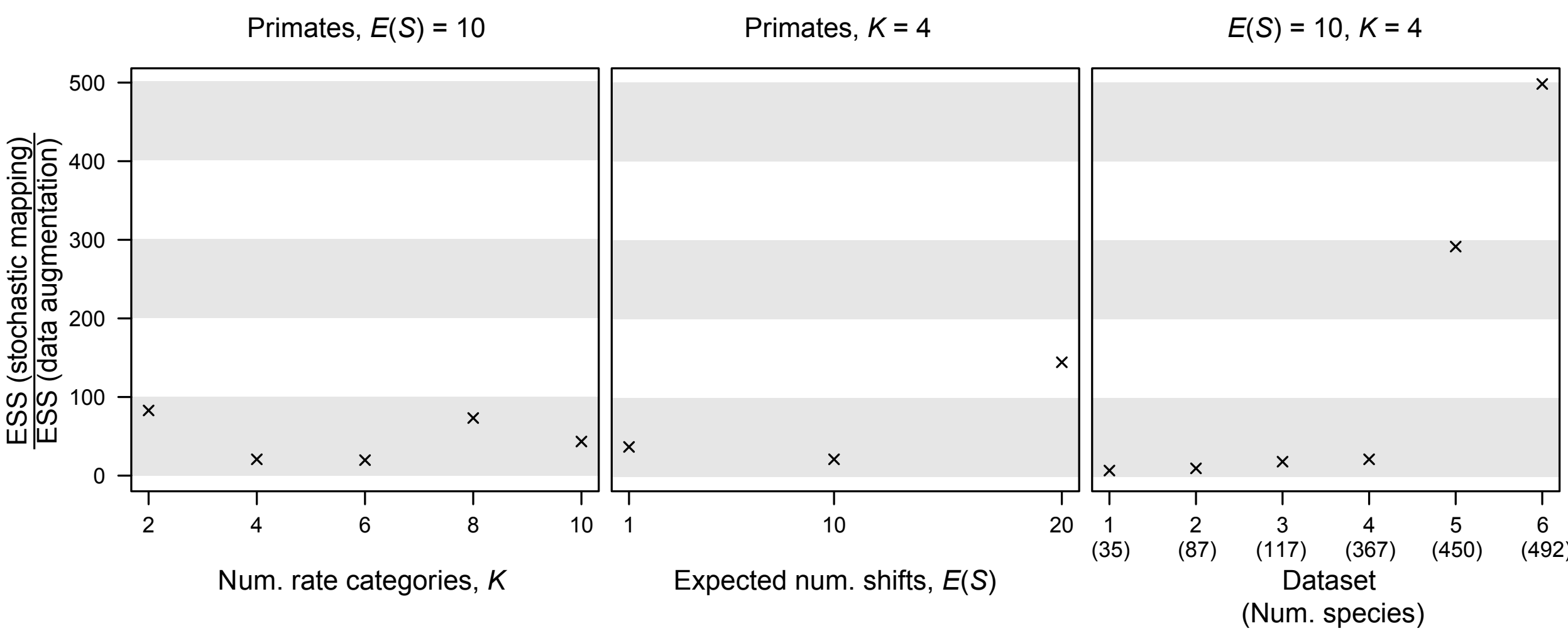

Figure S6: Comparing the computational efficiency of the data-augmentation and stochastic-mapping methods. We assessed the relative computational efficiency of our two BDS methods with analyses of six empirical datasets (Table S5). To this end, we compute a summary statistic as (average ESS for stochastic-mapping) $\div$ (average ESS for data augmentation). The left panel summarizes results for the primate phylogeny over a range of values for the number of rate categories, $K = \{2, 4, 6, 8, 10\}$, for a specific expected number of diversification-rate shifts, $E(S) = 10$. Conversely, the middle panel summarizes results for the primate phylogeny over a range of values for the expected number of diversification-rate shifts, $E(S) = \{1, 10, 20\}$, for a specific number of rate categories, $K = 4$. These results indicate that the stochastic-mapping method is $\approx 20 - 150$-fold more efficient than the data-augmentation method, but there appears to be no clear correlation between efficiency and the expected number of diversification-rate shifts or the number of rate categories. The right panel depicts results for the six datasets, where the expected number of diversification-rate shifts is $E(S) = 10$ and the number of rate categories is $K = 4$. These analyses again support the greater efficiency of the stochastic-mapping method, and suggest that the relative efficiency scales with the size of the dataset.

S3.3 Confirming correct coverage

Goal & Rationale—In Bayesian inference, the $X\%$ posterior credible interval of a parameter should contain ('cover') the true parameter value $X\%$ of the time [21, 22, 23]. For example, a clade that occurs in 95% of the trees sampled by an MCMC simulation has a posterior probability of 0.95, which we interpret to mean that there is a 0.95 probability that this clade is true, conditional on our study data and inference model. This interpretation assumes both that the model is valid (i.e., that the likelihood function correctly computes the probability of observing the data under the model and the prior distributions are correct) and that the MCMC algorithm is implemented correctly (i.e., that parameter values are sampled with frequency that is proportional to their posterior probability). Accordingly, failure to achieve this expected relationship between credible interval size and coverage probability indicates that either the likelihood function and/or MCMC algorithm are implemented incorrectly [23].

We validated our BDS likelihood function and MCMC algorithms using an approach called *simulation-based calibration* [22, 23]. In outline, this approach works as follows: (1) simulate R datasets from the prior of the model under consideration; (2) for each simulated dataset, use the MCMC algorithm under consideration to estimate the joint posterior probability distribution of the parameters, and; (3) compute coverage probabilities as the proportion of simulated replicates, R, where the credible interval of the estimated parameter value contains the true (simulated) parameter value. If the likelihood function of the model and the MCMC algorithm are correct, then these coverage probabilities will match the corresponding credible intervals [22, 24, 23]. We describe our coverage-validation experiments below.

Inference & Simulating Models—Performing simulation-based calibration to validate the coverage of our BDS approach requires that we first specify hyper/parameters of the BDS model in order to simulate datasets. Our previous validation experiments used fixed values for the BDS hyperparameters; i.e., for the mean and standard deviation of the lognormal speciation-rate prior ($\theta_\lambda, \sigma_\lambda$, respectively), and the extinction rate, μ. By contrast, we need to use a hierarchical BDS process for this validation experiment, i.e., specifying hyperpriors on the hyperparameters (rather than fixed values). Specifically, we specified hyperparameters of the lognormal speciation-rate prior as follows: we specified a lognormal hyperprior on the mean, θ_λ, and an exponential hyperprior on the standard deviation, σ_λ. As usual, we discretized the lognormal speciation-rate prior into K quantiles, where the speciation rate of the ith category, λ_i, was the median of the corresponding quantile, $p = i/K - 0.5/K$. Finally, we specified a lognormal hyperprior on the event-rate parameter, η, with a fixed mean of $\theta_\eta = 0.1$ and a fixed standard deviation of $\sigma_\eta = 0.58740$. These hyperparameter values were specified such that the 95% prior interval on η spanned one order of magnitude, which provided a reasonable number of diversification-rate shifts over the simulated trees. We used a uniform prior on the rate category at the root, Unif$(1, K)$. We list the BDS model parameters in Table S6.

Table S6: Model parameter names and values or prior distributions.

Parameter	X	$f(X)$
Mean speciation rate	θ_λ	Lognormal$(\ln(N/2.0)/2.0, 0.587405)$
Sd speciation rate	σ_λ	Exponential$(1.0/0.587405)$
Speciation rates	λ_i	qlnorm$(p, \theta_\lambda, \sigma_\lambda)$
Relative extinction rate	ϵ	Beta$(1, 1)$
Extinction rates	μ_i	$\epsilon \times \theta_\lambda$
Shift rate	η	Lognormal$(0.1, 0.587405)$
Root category	r	Unif$(1, K)$

N is the number of species in the simulated tree.

Data—We simulated a total of 1000 trees from the prior of the BDS process described above (Table S6). In our simulations, we used $K = 4$ diversification-rate categories and conditioned on a tree size of 200 tips. This tree size strikes an acceptable compromise between statistical power (big trees) and computational tractability (small trees). We simulated forward in time under the BDS process until a tree had 201 tips, and then randomly trimmed the tree on the interval between the origin of the 200th and 201st tip.

Analyses—We performed analyses of each simulated tree under the BDS process (as specified in Table S6) using the stochastic-mapping algorithm implemented in `RevBayes`. As when we simulated the trees, we assumed $K = 4$ discrete rate categories. We provide the scripts necessary to perform these analyses (with details of the proposal mechanisms, chain length, etc.) in the `5_coverage` directory of our GitHub repository: https://github.com/hoehna/birth-death-shift-analyses.

Results—To assess the coverage probability of our BDS approach, we first defined 20 credible intervals of equal width, 0.5, ranging from $0 - 1$. We then calculated coverage probabilities for a given parameter as the proportion of cases across the 1000 simulations where the credible interval contained the true (simulated) value. If the likelihood function of our BDS model correctly computes the probability of observing a given tree, and our MCMC inference algorithms are implemented correctly, then the coverage probabilities should be equal to their corresponding credible intervals [22, 24, 23]. Indeed, the results of our simulation-based calibration experiments confirm that the coverage probabilities for our BDS method are correct (Figure S7).

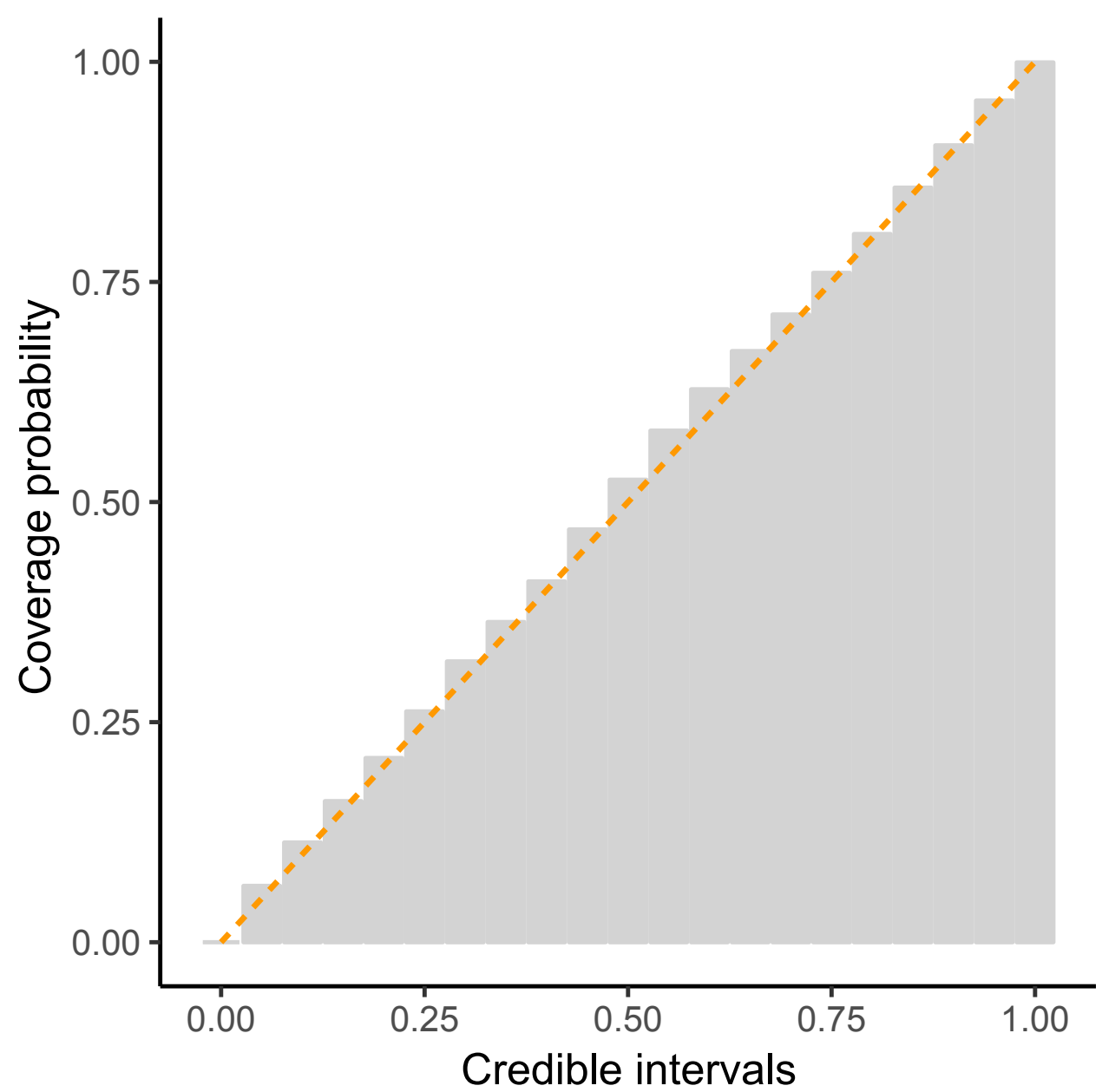

Figure S7: Validating coverage probabilities for our BDS method by means of simulation-based calibration. We simulated 1000 trees from the prior of the BDS process, and then estimated parameters of the same BDS process from each simulated tree. We first defined 20 credible intervals of equal width, $\{0.00-0.05, 0.05-0.10, 0.10-0.15, \ldots, 0.95-1.0\}$ (x-axis). We then calculated coverage probabilities (y-axis) as the proportion of cases across the 1000 simulations where the credible interval for a parameter contained the true (simulated) value (here, we plot estimates of branch-specific net-diversification rates). If the likelihood function of our BDS model correctly computes the probability of observing a given tree, and our MCMC inference algorithms are implemented correctly, then the coverage probabilities should fall along the dashed line.

S4 Characterizing the Statistical Behavior of the BDS Method

S4.1 Simulation Study: Testing the Accuracy of Branch-Specific Diversification-Rate Estimates

Goal—Having validated the theory and implementation of the BDS process, we conducted a simulation study to assess the accuracy of parameter estimates and branch-specific rate estimates. While a correct derivation and implementation are necessary for likelihood based inference, parameter estimation can be biased for small sample sizes, or even be non-identifiable [25]. Our aim for this simulation study was to explore the ability of the birth-death-shift model to infer branch-specific diversification rates as well as hyperparameters of the model. Some parameters may be easier to estimate than others, for example we expect that branch-specific speciation rates are easier to infer than branch-specific extinction rates [4].

Data—We simulated 100 trees under the birth-death-shift process forward in time with parameters chosen so that there were relatively few, relatively large diversification-rate shifts. We assumed that both speciation and extinction rates varied, each within $K = 4$ discrete rate categories. Note that we conditioned in our simulations that trees must contain between 20 to 10,000 tips, as small trees do not contain sufficient information and large trees are computationally slow. Both the speciation and extinction rates were allowed to vary. The true parameter values are described in Table S7. On average, our simulated trees had $N \approx 379$ tips (similar to the empirical primates phylogeny). We show the distribution of the number of taxa in the simulated trees in Figure S8, and the 100 simulated trees with branches colored by net-diversification rates in Figure S9.

Analyses—We performed analyses of each simulated tree under the BDS process with priors as specified in Table S7) in `RevBayes`. As when we simulated the trees, we assumed $K = 4$ discrete rate categories for the speciation- and extinction-rate distributions. We provide the scripts necessary to perform these analyses in the `10_simulation_study` directory of our GitHub repository: https://github.com/hoehna/birth-death-shift-analyses.

Table S7: Model parameter names, true values (for simulation) and prior distributions $f(X)$ (for inference).

Parameter	X	True value	$f(X)$
Root age	t_r	71.3547	–
Mean speciation rate	θ_λ	0.05	Lognormal($\ln(N/2.0)/2.0, 0.587405$)
Sd speciation rate	σ_λ	2×0.587405	Exponential(1.0/0.587405)
Speciation rates	λ_i	qlnorm($p, \theta_\lambda, \sigma_\lambda$)	–
Mean extinction rate	θ_μ	0.0375	Lognormal($\ln(N/2.0)/2.0, 0.587405$)
Sd extinction rate	σ_μ	0.587405	Exponential(1.0/0.587405)
Extinction rates	λ_i	qlnorm($p, \theta_\mu, \sigma_\mu$)	–
Shift rate	η	0.0025	Lognormal(0.1, 0.587405)
Root category	r	Unif($1, K^2$)	Unif($1, K^2$)

N is the number of species in the simulated tree.

Results—The hyperparameters were estimated relatively well (Figure S10). The prior mean of the speciation rates, θ_λ, was estimated more robustly than the prior mean of the extinction rate, θ_μ. Specifically, uncertainty in the prior mean of the extinction rates θ_μ was higher, although the posterior mean was close to the true value. The prior standard deviation of the speciation rates, σ_λ, and extinction rates, σ_μ, were slightly underestimated. Again, the uncertainty in the extinction-rate hyperparameter was higher; this has previously been observed [4] and is expected because we have more information about speciation events than extinction events.

The shift rate, η, was more challenging to estimate. Because the shift rate η is correlated with the degree of diversification-rate variation (σ_λ and σ_μ), when the simulated tree does not experience a rate shift, there will be little information in the data to infer these parameters. Interestingly, the standard deviation of the speciation rates, σ_λ, was most often estimated to be very small, which has the consequence that rate shifts have no or only minor effect. This may be due to the exponential prior distribution on σ_λ.

Our general observations of our simulation-study results conform to four scenarios, which we illustrate with simulated trees 2, 20, 35 and 52. We selected these trees as representatives for illustration, but the overall

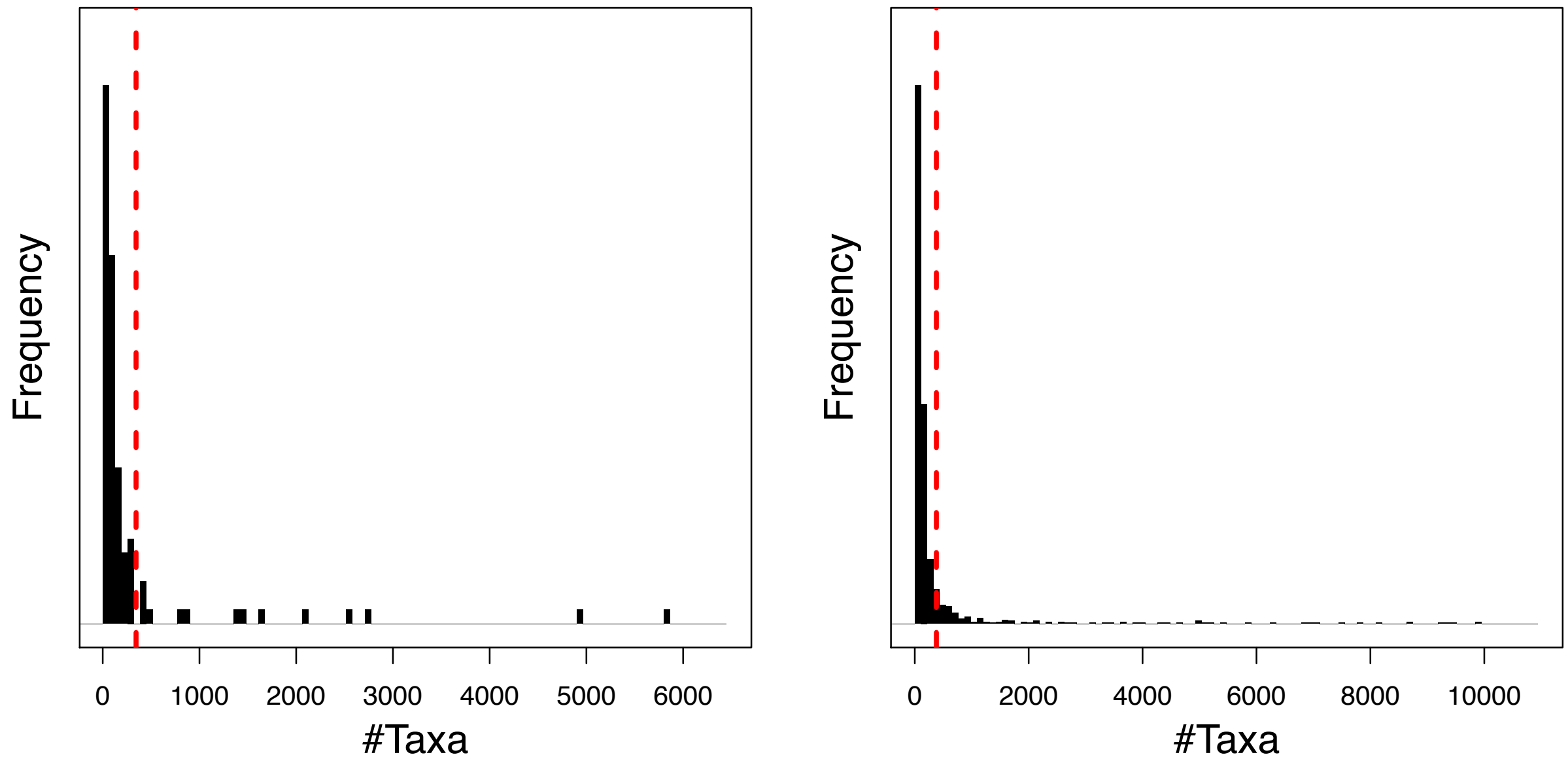

Figure S8: Distribution of number of tips for 100 simulated trees (left) and 1000 simulated trees (right) under the birth-death-shift process. The red dashed line shows the mean of $\bar{N} = 379.292$. We note that this distribution is much more skewed than the distribution of number of tips under a lineage-homogeneous birth-death process.

pattern can be confirmed from Figures S9 and 8 (main text). (1) The simulated tree did not experience a rate shift. In this case our method correctly infers no rate shift and the branch-specific diversification rates match (Figure S11). Interestingly, the model interpreted the absence of a rate as a very narrow base distribution, i.e., with a very low standard deviation, σ_λ or σ_μ, instead of a low shift rate. Thus, we inferred that there could have been rate shifts, but they had no discernible impact. (2) The best-case scenario for our BDS method corresponds to a rate shift that gives rise to a large subtree. In that case, our method recovered all hyperparameter and branch-specific diversification rates accurately (Figure S12). (3) If the simulated tree was, by chance, relatively small, then our method produced biased parameter estimates and was not able to detect any rate shifts (Figure S13). However, it was previously shown that diversification rates can be biased for small trees [26]. We therefore suggest that our method not be applied to small trees. (4) Rate shifts that occurred on terminal branches or branches leading to small subtrees could not be detected (Figure S14). This lack of power is neither surprising nor worrisome; there simply is not sufficient information in a single lineage to estimate a separate set of diversification rates.

In summary, our simulation study demonstrates that our method does not detect spurious rate shifts and behaves rather conservatively. Overall, branch-specific diversification rates can be estimated reliably, even if we choose wide and uninformative prior distributions. If the overall model assumptions are not violated, then we can robustly infer parameters under the BDS model without identifiability issues.

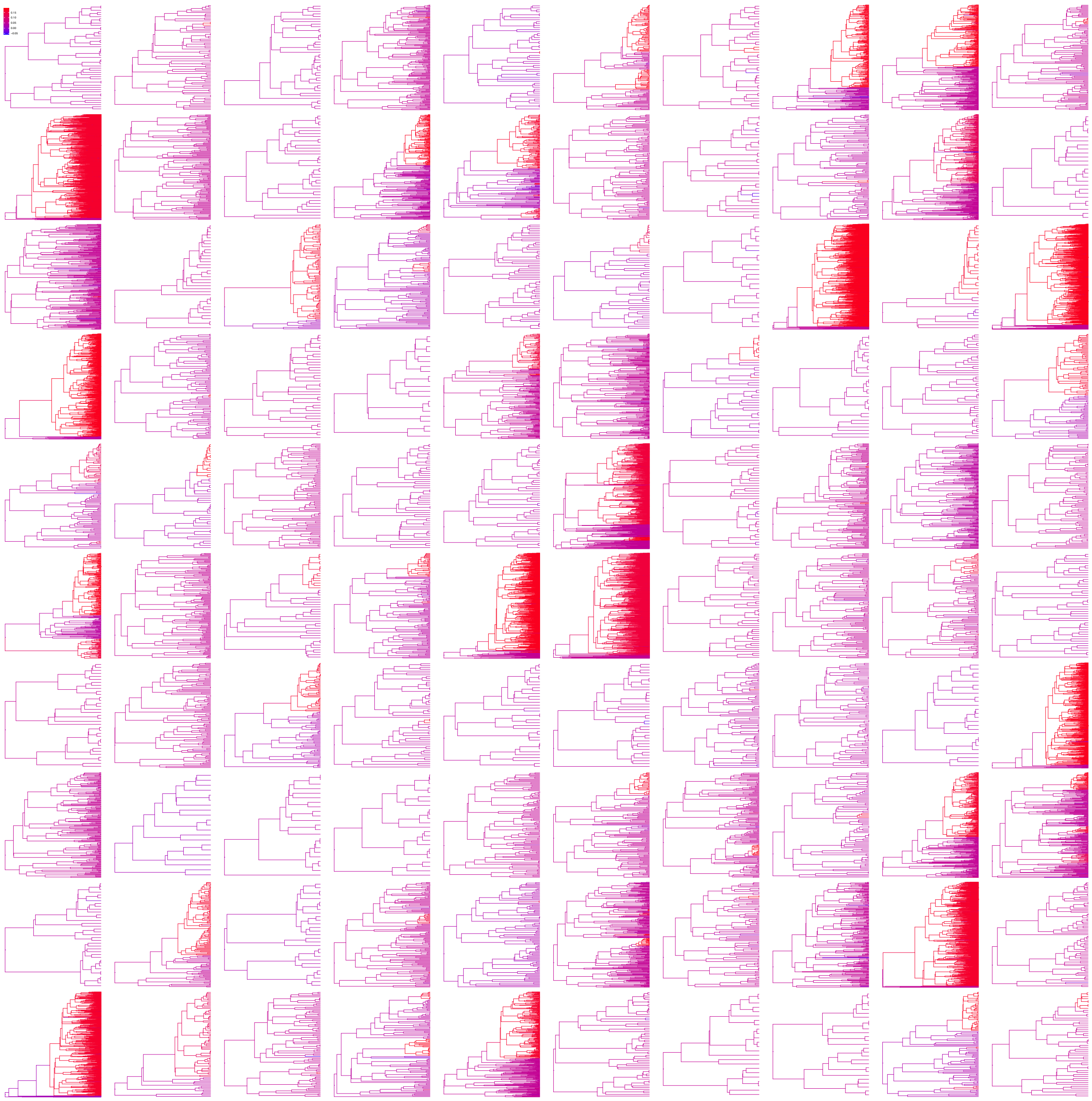

Figure S9: 100 simulated trees under the birth-death-shift process. Branches are colored according to the average net-diversification rate.

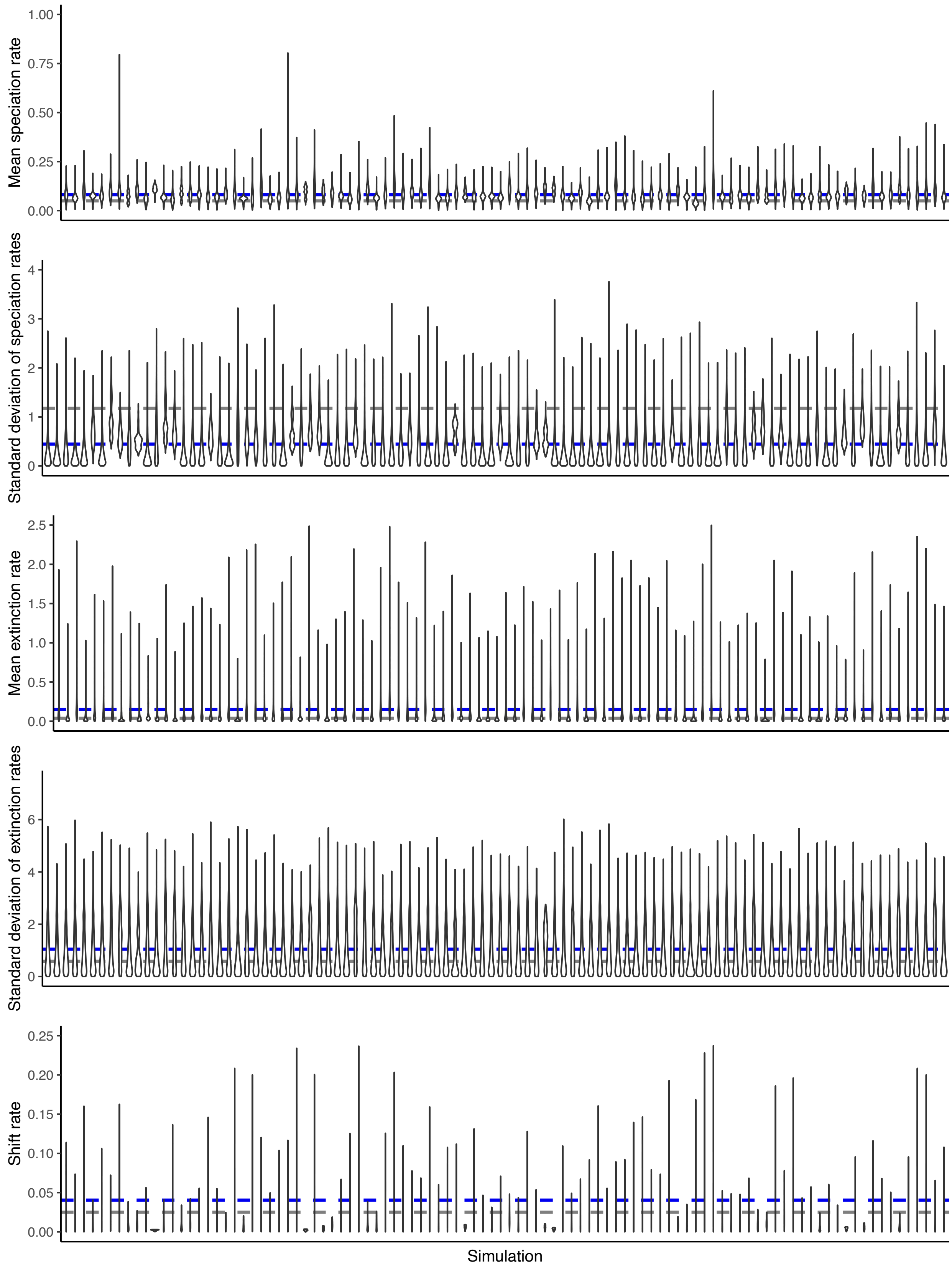

Figure S10: Posterior distributions of the hyperparameters for all 100 simulated trees. The gray dashed line shows the true value and the blue dashed line shows the overall mean posterior estimate averaged over all simulations.

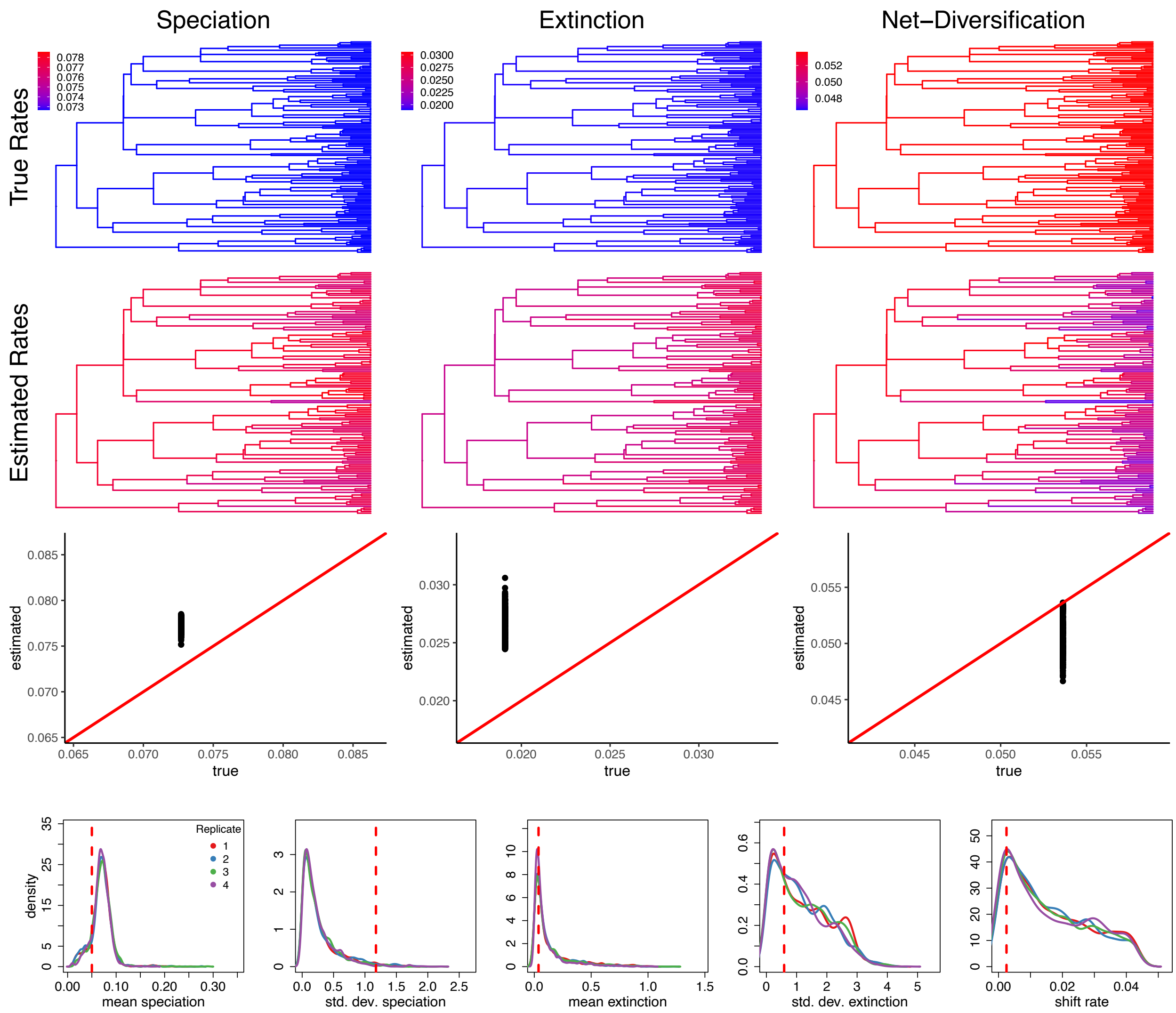

Figure S11: Estimated branch-specific speciation rates for simulated tree 52. This examples shows a simulation that did not experience a rate shift. Overall, the branch-specific diversification rates are estimated very well, although slightly overestimated. Note, however, that the estimated mean rates and the true rate (dashed red line) values fall well within the 95% credible intervals (bottom row). Interestingly, the parameter for the speciation-rate variation, sd_λ, is strongly underestimated. Thus, all speciation rates are almost equal. The shift rate cannot be estimated well, because the speciation rate variation was underestimated.

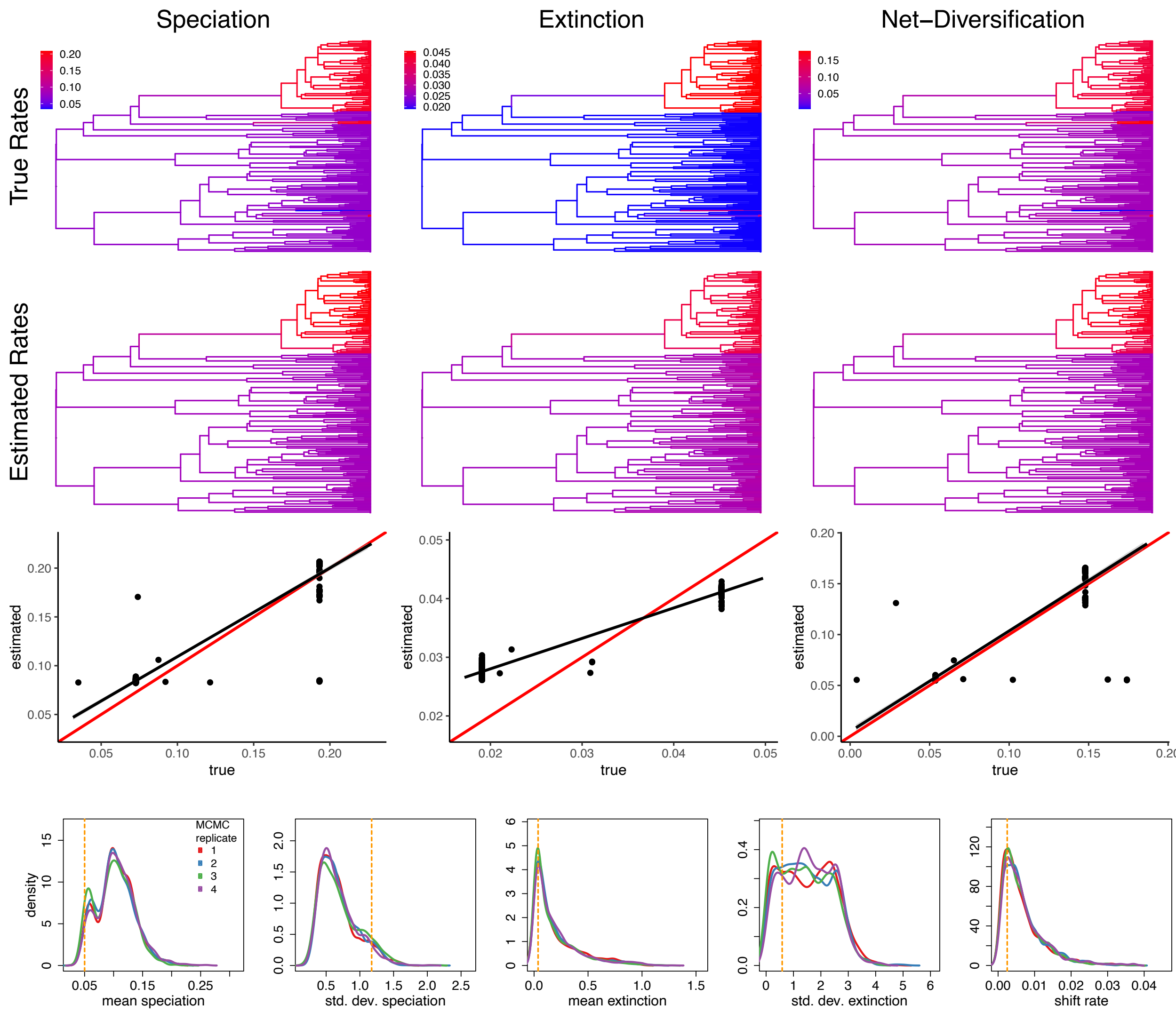

Figure S12: Estimated branch-specific speciation rates for simulated tree 35. This examples shows a best-case scenario, where the tree experienced a rate shift that gave rise to a large subtree. The branch-specific diversification rates and their hyperparameters are very well estimated.

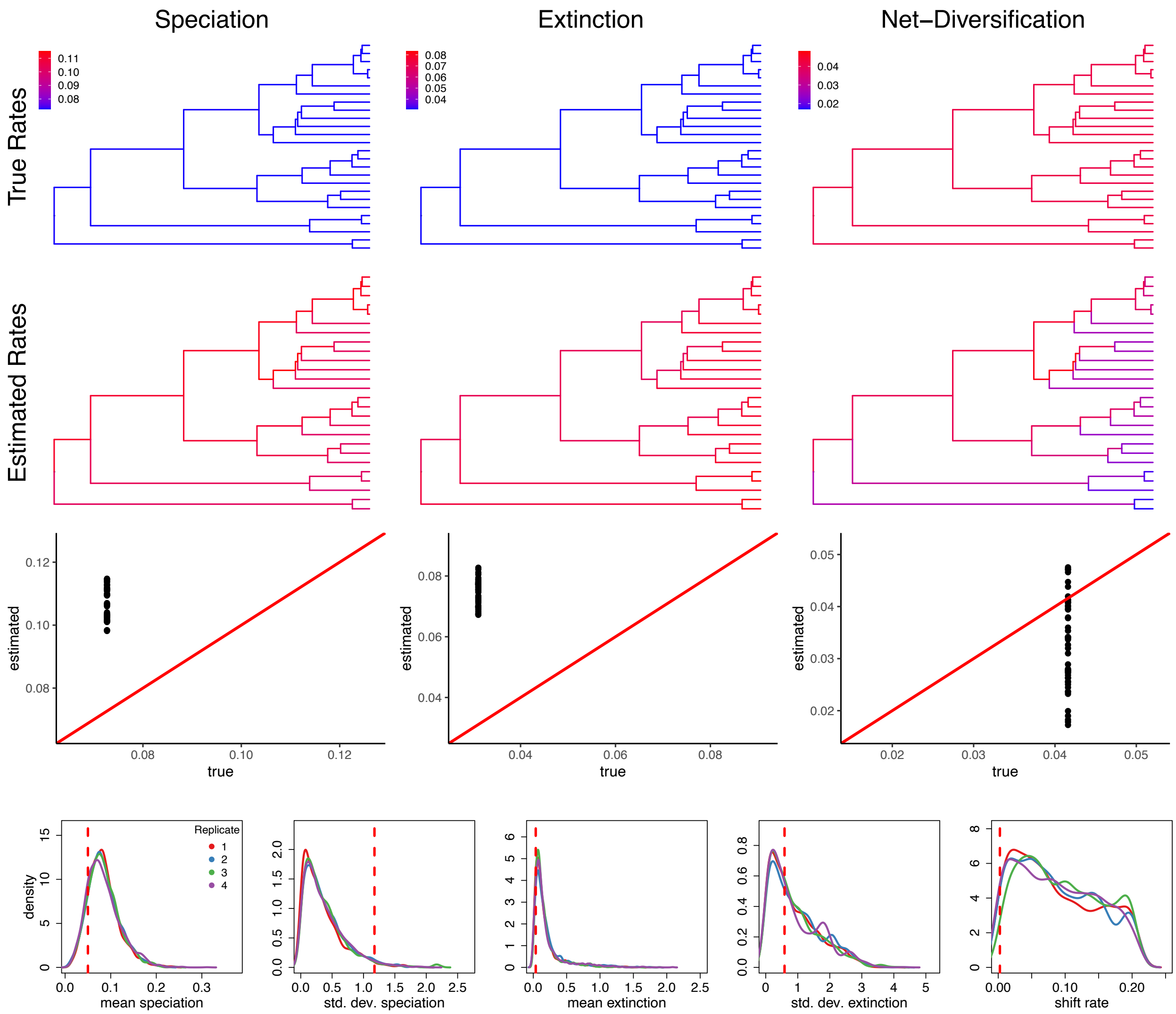

Figure S13: Estimated branch-specific speciation rates for simulated tree 20. This examples shows a simulated tree that is comparably small. Because of the small tree size, neither the branch-specific diversification rates or their hyperparameters are well estimated.

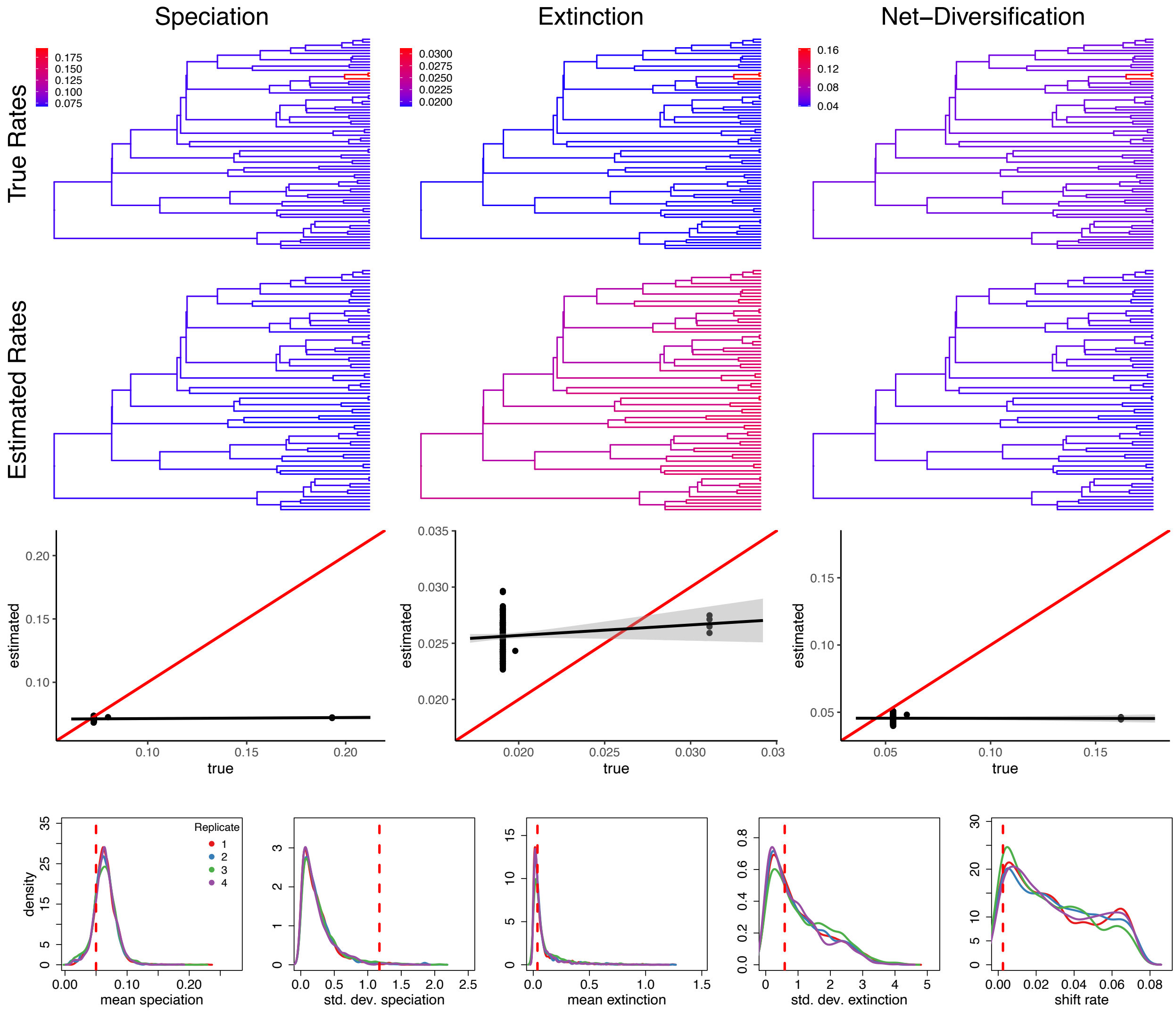

Figure S14: Estimated branch-specific speciation rates for simulated tree 2. This examples shows a tree that experienced a single rate shift leading to very small subtree with only two sampled species. Because the subtree with a distinct diversification-rate category is too small, we cannot infer this rate-shift event. The resulting hyperparameter estimates are qualitatively identical to simulations without rates shifts.

S5 Empirical Analyses of Primates

We performed a series of analyses on an empirical dataset—a dated phylogeny of 367 primate species [13]—with four main goals: (1) to demonstrate how we can use Bayes factors to select among competing diversification models; (2) to assess the impact of discretization bias on estimates of branch-specific diversification rates; (3) to explore the impact of the event-rate prior, η, on (a) estimates of the inferred number of diversification-rate shifts, and (b) estimates of branch-specific diversification rates, and; (4) to explore our ability to estimate hyperparameters of the BDS process. We describe each of these experiments below.

S5.1 Comparing lineage-diversification models using Bayes factors

Goal & Rationale—Our implementation of the BDS process in `RevBayes` allows us to specify a ginormous diversity of diversification models. For example, we can specify four major classes of BDS models where a diversification-rate shift: (1) exclusively impacts speciation rates; (2) exclusively impacts extinction rates; (3) impacts both speciation and extinction rates, or; (4) impacts net-diversification rates. For each of these four classes of BDS models, in turn, we can specify an array of submodels that differ with respect to the probability distributions specified for the speciation and/or extinction rates (such as the lognormal, gamma, exponential distributions). Moreover, for each specific BDS class/base-distribution, we can specify a number of submodels that differ with respect to how we model the hyperparameters of the base distributions. For example, in the BDS model class where diversification-rate shifts impact both speciation and extinction rates, we may specify lognormal base distributions for the diversification rates: we can further specify different submodels that differ with respect to how we treat the mean and standard-deviation parameters of the lognormal base distributions ($\theta_\lambda, \sigma_\lambda$ and θ_μ, σ_μ), respectively); e.g., we may use fixed values for these parameters or treat them as random variables by specifying hyperpriors (lognormal, uniform, exponential, etc.).

The large diversity of BDS models affords two main advantages: (1) identify a model that provides good fit to our dataset and thus should provide more reliable and accurate estimates of branch-specific diversification rates. (2) hypothesis testing to compare two competing models to test hypotheses about various features of diversification-rate variation. Here, we demonstrate comparing the relative fit of two competing models—the constant-rate birth-death process to the BDS process—to the primate tree. This corresponds to testing the hypothesis that rates of diversification in the primate tree are constant across linages. We compare the relative fit of two competing diversification models to a given dataset using the Bayes factor for the two candidate diversification models [27].

Data—We performed these model selection analyses on the dated phylogeny of primates published by Springer et al. [13], where we treated this phylogeny as an observation.

Analyses—We estimated marginal likelihoods to compute the Bayes factor for two models: a constant-rate birth-death process and a birth-death-shift process. We provide the parameters and prior distributions for both models in Table S8. We estimated the marginal likelihood using stepping-stone sampling [28] using the parallel power posterior sampler in `RevBayes` [29], We set up 100 power posteriors, and for each we performed 500 MCMC steps. We provide the scripts necessary to perform these analyses in the `9_bayes_factor` directory of our GitHub repository: https://github.com/hoehna/birth-death-shift-analyses.

Results—Our estimates of marginal-likelihoods to compute Bayes factors for the competing models indicate a clear preference for the birth-death-shift model with a log-transformed Bayes factor of $\log_e(\mathrm{BF}_{\mathrm{VC}}) = 10.8$. This Bayes factor is interpreted as 'decisive' [30], 'extreme' [31], or 'very strong' [27] support for the birth-death-shift model over the constant-rate model.

Table S8: Model parameter names and values or prior distributions.

Model	Parameter	X	$f(X)$
Birth-Death-Shift:			
	Mean speciation rate	θ_λ	Loguniform$(10^{-6}, 100)$
	Sd speciation rate	σ_λ	Exponential(1.0/0.587405)
	Speciation rates	λ_i	qlnorm$(p, \theta_\lambda, \sigma_\lambda)$
	Extinction rates	μ_i	Loguniform$(10^{-6}, 100)$
	Shift rate	η	$2/TL$
	Number of categories	k	10
	Root category	r	Unif$(1, K)$
Constant-rate Birth-Death:			
	Speciation rate	λ	Loguniform$(10^{-6}, 100)$
	Extinction rate	μ	Loguniform$(10^{-6}, 100)$

TL is the length of the primate tree.

S5.2 Impact of discretization on branch-specific diversification-rate estimates

Goal & Rationale—In our implementation, we used a discretized distribution for mathematical and computation convenience to approximate a continuous based distribution from which the diversification rates are drawn after a rate shift event. Thus, here we assess the robustness of the diversification-rate estimates when changing the number of diversification-rate categories, K. For this analysis we only used the numerical-integration approach because: (1) we've previously established that both methods yield identical diversification-rate estimates, and; (2) the we've previously established the higher computational efficiency of the numerical-integration approach. We specified the model as follows. We specified a log-uniform distribution on both the hyper-parameter m_λ and m_μ. Then, we computed the speciation rates for i-th category as the quantile of a lognormal distribution with $p = i/k - 0.5/k$. The extinction rates for all categories were set to be equal to m_μ. As before, the shift rate was arbitrarily fixed to $\eta = 2/TL$ so that we expect two diversification-rate shifts. We varied the number of diversification rate categories, $K = \{4, 6, 8, 10, 12, 20\}$.

Data—We performed these model selection analyses on the dated phylogeny of primates published by Springer et al. [13], where we treated this phylogeny as an observation.

Analyses— The scripts for performing these analyses can be found in the directory 6_prior_sensitivity_num_cats of our GitHub repository https://github.com/hoehna/birth-death-shift-analyses. The `Rev` scripts provide all of the information regarding the MCMC settings (i.e., how many replicate MCMC runs were performed for how many cycles each and which moves were used) and model settings used for these analyses. Additionally, in Table S9 we provide a summary of all model parameters.

Table S9: Model parameter names and values or prior distributions. TL represents the length of tree for this study group.

Parameter	X	$f(X)$
Mean speciation rate	m_λ	Uniform$(0, 10^2)$
Mean extinction rate	m_μ	Uniform$(0, 10^2)$
Sd speciation rate	sd_λ	0.587405
Speciation rates	λ_i	qlnorm$(p, m_\lambda, sd_\lambda)$
Extinction rates	μ_i	m_μ
Shift rate	η	$2/TL$
Number of categories	k	$\{4, 6, 8, 10, 12, 20\}$
Root category	r	Unif$(1, k)$

Results—The plot summarizing the results of these analyses is shown in Figure S15. Our results show that $k = 6$ speciation rate categories might be a sufficient approximation of the full continuous distribution to obtain robust branch-specific speciation rate estimates (see also Kopperud and Höhna [4]).

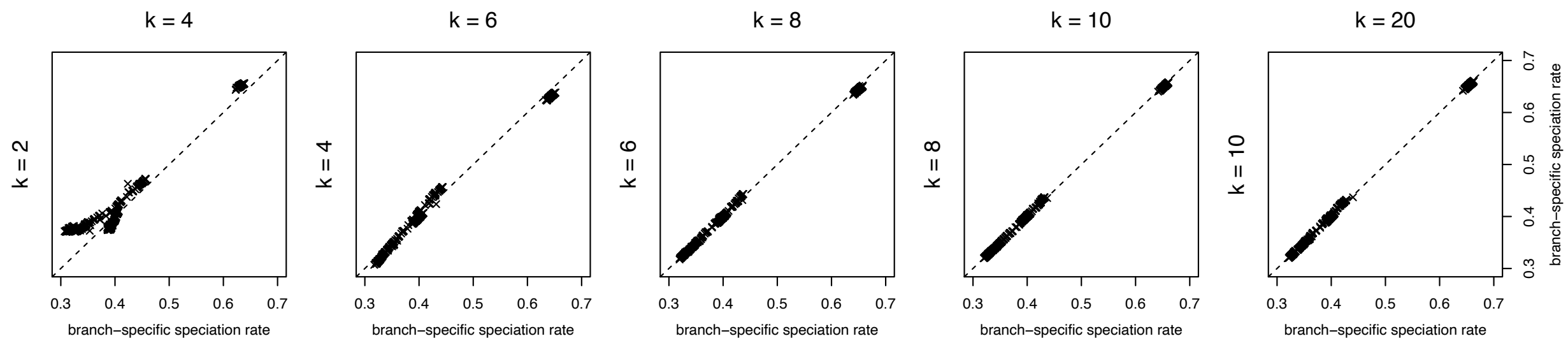

Figure S15: Comparison of branch-specific rate estimates for different numbers of diversification-rate categories. We estimated the posterior mean branch-specific speciation rate for each branch of the primate tree where the number of rate categories was set to $k = \{2, 4, 6, 8, 10, 20\}$. We then compared the mean estimates of the rates between adjacent pairs of the number of diversification-rate categories. For small numbers of diversification-rate categories, the branch-rate estimates are quite different between adjacent settings. However, as the number of categories increases, the branch-specific diversification-rate estimates converge toward stable estimates.

S5.3 Impact of the event-rate prior on parameter estimates

Impact of the event-rate prior on estimates of the number of diversification-rate shifts

Goal & Rationale—In principle, the number and location of diversification rate shifts is one of the primary goals for a birth-death-shift analysis. However, the number of shifts is at best weakly identifiable [14] and the shift rate itself can have a strong impact on the inferred number of shifts [4]. In principle, there could be many small shifts or few large shifts. Here, we assess the prior sensitivity of the estimated number of diversification-rate shifts. This exploration will show on an empirical example the actual extend of prior sensitivity, and its impact on our ability to estimate the number of diversification rate shifts.

Data—Our exploration of the prior sensitivity of the number of rate shifts on the number of inferred rate shifts used the dated phylogeny of primates published by Springer et al. [13], where we treated this phylogeny as an observation.

Analyses— Thus, we performed analyses over a range of values for the expected number of shifts. The setup of these analyses was otherwise almost identical to that of the previous analysis, except that here we fixed the number of diversification-rate categories $k = 10$ and changed the shift rate to $\eta = E(S)/TL$ where $S = \{1, 5, 10, 20, 50, 100\}$, see Table S10 for all model parameters and their prior distributions or fixed values. The scripts for performing these analyses can be found in our GitHub repository https://github.com/hoehna/birth-death-shift-analyses in the directory 7_prior_sensitivity_shifts. The `Rev` scripts provide all of the information regarding the MCMC settings (i.e., how many replicate MCMC runs were performed for how many cycles each and which moves were used) and model settings used for these analyses.

Table S10: Model parameter names and values or prior distributions. TL represents the length of tree for this study group.

Parameter	X	$f(X)$
Mean speciation rate	m_λ	Uniform$(0, 10^2)$
Mean extinction rate	m_μ	Uniform$(0, 10^2)$
Sd speciation rate	sd_λ	0.587405
Speciation rates	λ_i	qlnorm$(p, m_\lambda, sd_\lambda)$
Extinction rates	μ_i	m_μ
Shift rate	η	$E(S)/TL$
Number of categories	k	10
Root category	r	Unif$(1, k)$

Results— Our results are shown in Figure 10A of the main manuscript and replicated in Figure S16. We observe a very strong prior sensitivity of the prior on the number of diversification rate shifts on the posterior distribution of the number of diversification rate shifts. The posterior distribution and prior distribution are

not exactly the same, indicating that the model is identifiable with respect to the number of diversification rate shifts, however, the information in the data is weak to overwhelm the prior distribution.

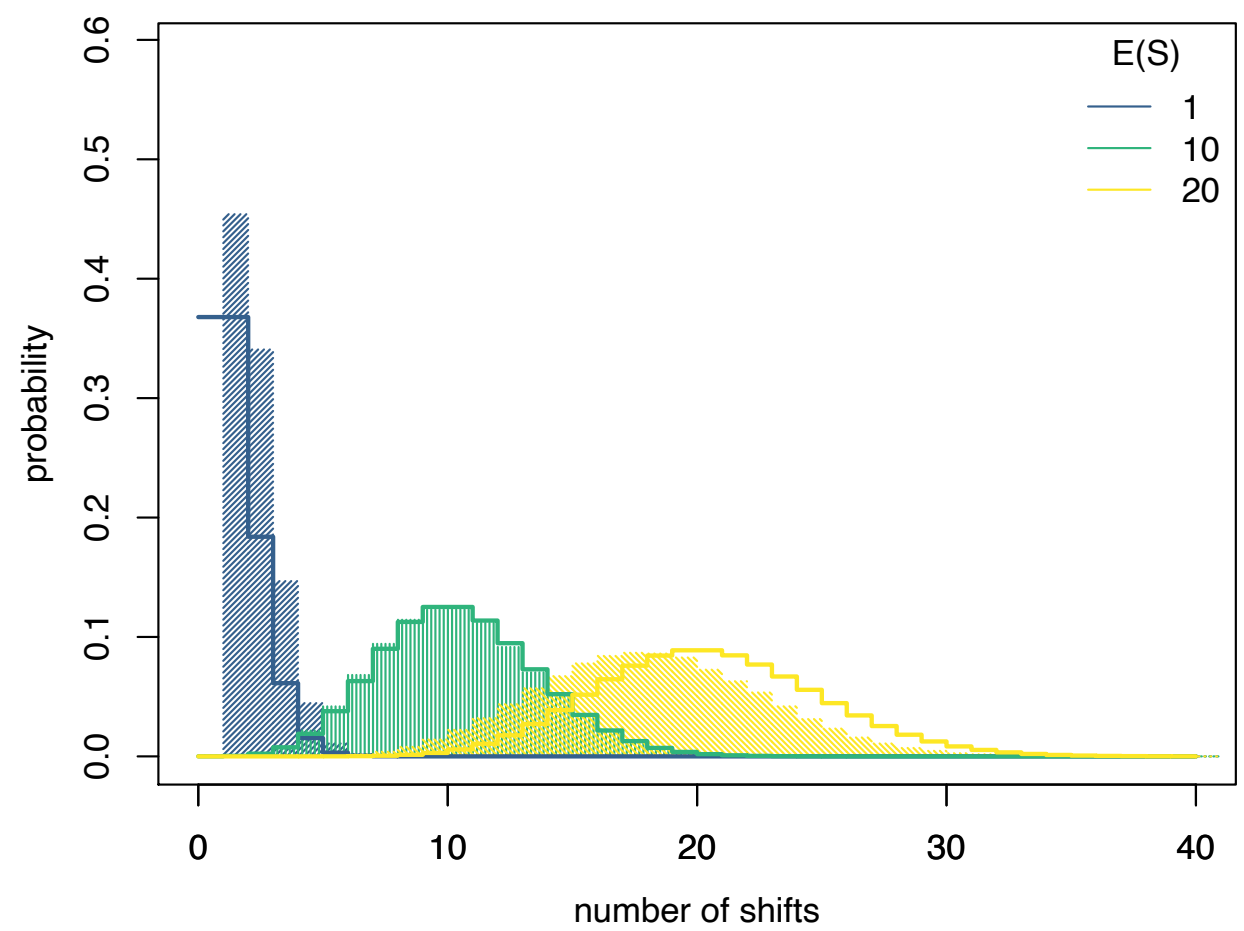

Figure S16: Comparison between the prior number of diversification-rate shifts and the posterior number of diversification-rate shifts for different shift-rate priors. We estimated the posterior number of diversification-rate shifts (shaded bars) in the primate phylogeny under three different shift-rate priors, with the prior on η specified so that the prior expected number of shifts under a Poisson process, $E(S)$, was 1, 10, or 20 (solid lines). The posterior number of diversification-rate shifts is very sensitive to these prior settings although not exactly matching the prior distributions.

Impact of the event-rate prior on estimates of branch-specific diversification rates

Goal & Rationale—In the above exploration we showed that the estimated number of diversification rates is extremely sensitive to the prior distribution on the number of diversification rate shifts. However, the impact of the number of shifts on the estimated branch-specific diversification rates is less explored. Here, we actually used the output produced from the previous analysis: *Prior Sensitivity of the Estimated Number of Diversification-Rate Shifts*. Hence, we used the same `Rev` scripts and model were as shown in Table S10.

Results—Our results show that there is some impact of the prior distribution of the number of diversification rate shifts on the branch-specific diversification rate estimates (Figure S17). This impact is further illustrated in Figure 10B of the main text. While the general pattern is less impacted, i.e., we still see clear distinctions in diversification rates in the New World Monkeys clade, the actual branch-specific diversification rates change. Nevertheless, the branch-specific diversification are less sensitive to the prior distribution on the number of diversification rate shifts as the estimated number of diversification rate shifts itself.

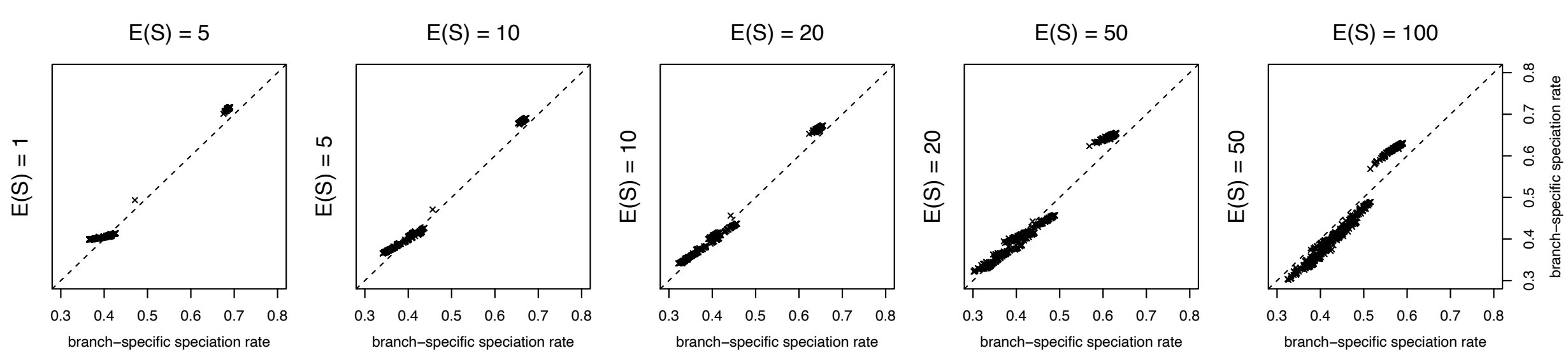

Figure S17: Comparison of branch-specific speciation-rate estimates between different priors on the expected number of diversification-rate shifts. We estimated the posterior mean speciation rate for each branch of the primate tree under different shift-rate priors, with the prior on η specified so that the prior expected number of rate-shift events under a Poisson process, $E(S)$, was 1, 10, 20, 50 or 100. Despite the estimated number of diversification-rate shifts being prior sensitive (Figure S5), the branch-specific speciation-rate estimates are relatively robust to the prior on the expected number of diversification- rate shifts.

S5.4 Estimating hyperparameters of the BDS process

Goal & Rationale— In our final analysis, our aim is to assess whether there is any information in estimating the hyper-parameters of the diversification-rate base distributions. In several of the previous analyses, we fixed the mean speciation rate to $(N-2)/TL$, which may be viewed as a reasonable guess because it corresponds to the maximum-likelihood estimate under a pure-birth process. However, this 1empirical Bayesian' approach also uses the data twice: once to inform he prior, and then a second time to estimate the posterior. Thus, we specified four hyperprior distributions: (1) we specified a log-uniform distribution on the hyper-parameter m_λ, (2) we specified a log-uniform distribution on the hyper-parameter m_μ, (3) we specified an exponential distribution on the hyper-parameter sd_λ, and; (4) we specified a uniform distribution on the shift rate η. The exact settings for these hyperprior distributions and all other model parameters can be found in Table S11.

Data—We performed the hyperprior analysis on the dated phylogeny of primates published by Springer et al. [13], where we treated this phylogeny as an observation.

Analyses— The scripts for performing these analyses can be found in the directory 8_hyperprior in our GitHub repository https://github.com/hoehna/birth-death-shift-analyses. The `Rev` scripts provie all of the information regarding the MCMC settings (i.e., how many replicate MCMC runs were performed for how many cycles each and which moves were used) and model settings used for these analyses.

Table S11: Model parameter names and values or prior distributions. TL represents the tree length of this study phylogeny.

Parameter	X	$f(X)$
Mean speciation rate	m_λ	Uniform$(0, 10^2)$
Mean extinction rate	m_μ	Uniform$(0, 10^2)$
Sd speciation rate	sd_λ	Exponential(1.0/0.587405)
Sd extinction rate	sd_μ	Exponential(1.0/0.587405)
Speciation rates	λ_i	qlnorm$(p, m_\lambda, sd_\lambda)$
Extinction rates	μ_i	qlnorm(p, m_μ, sd_μ)
Shift rate	η	Uniform$(0.0, 100.0/TL)$
Number of categories	k	10
Root category	r	Unif$(1, k)$

Results—Our results clearly show that we can infer the hyper-parameters of the birth-death-shift process (Figure S18). The posterior distributions deviate from the prior distributions. However, there might be weak information in the data as some posterior distributions are rather wide. Interestingly, even the shift rate has a non-uniform posterior distribution, indicating that the shift rate is not completely driven by the prior distribution and therefore also the estimated number of diversification rate shifts.

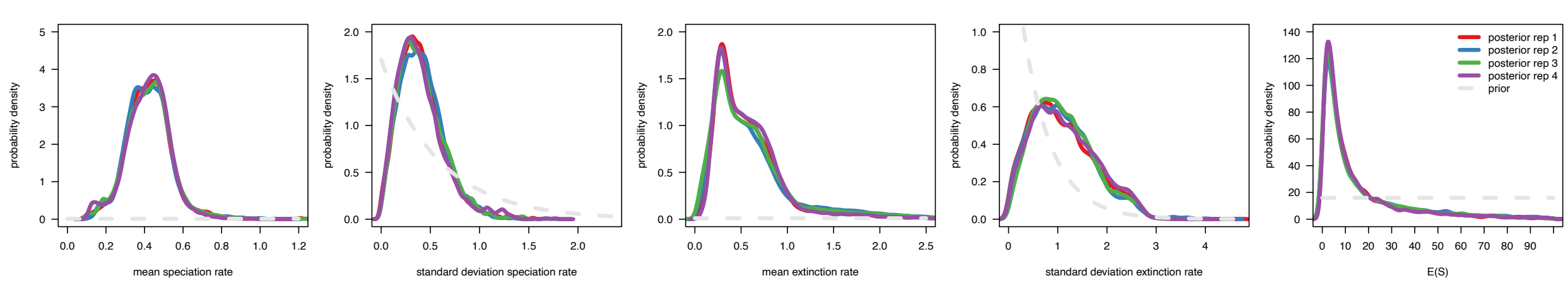

Figure S18: Estimating hyperparameters under the hierarchical BDS process model. We estimated the mean (m_λ and m_μ) and standard deviation (sd_λ and sd_μ) of the lognormal base distribution for the speciation rate. Additionally, we estimated the shift-rate η (right panel; showing the expected number of shifts, $E(S)$, for an intuitive interpretation). We used the following prior distributions: $m_\lambda \sim$ Unif(0, 100), $sd_\lambda \sim$ Exp(1.0/0.587405), $m_\mu \sim$ Unif(0, 100), $sd_\mu \sim$ Exp(1.0/0.587405), and $E(S) \sim$ Unif(0, 100). The posterior distributions (black solid lines) show clear departure from the corresponding prior distributions (light-gray dashed lines). The four colors show different MCMC replicates. The agreement between the MCMC replicates shows the convergence of the MCMC runs.

References

[1] Joelle Barido-Sottani, Timothy G Vaughan, and Tanja Stadler. A multitype birth-death model for Bayesian inference of lineage-specific birth and death rates. *Systematic Biology*, 69(5):973–986, 2020.

[2] Sebastian Höhna, Michael J. Landis, Tracy A. Heath, Bastien Boussau, Nicolas Lartillot, Brian R. Moore, John P. Huelsenbeck, and Fredrik Ronquist. RevBayes: Bayesian phylogenetic inference using graphical models and an interactive model-specification language. *Systematic Biology*, 65(4):726–736, 2016. doi: 10.1093/sysbio/syw021.

[3] Daniel L Rabosky. Automatic detection of key innovations, rate shifts, and diversity-dependence on phylogenetic trees. *PLoS One*, 9(2):e89543, 2014.

[4] Bjørn T Kopperud and Sebastian Höhna. Phylogenetic estimation of branch-specific shifts in the tempo of origination. *Systematic Biology*, page syaf041, 2025.

[5] W.P. Maddison, P.E. Midford, and S.P. Otto. Estimating a binary character's effect on speciation and extinction. *Systematic Biology*, 56(5):701–710, 2007.

[6] R.G. FitzJohn, W.P. Maddison, and S.P. Otto. Estimating trait-dependent speciation and extinction rates from incompletely resolved phylogenies. *Systematic Biology*, 58(6):595–611, 2009.

[7] Richard G. FitzJohn. Diversitree: comparative phylogenetic analyses of diversification in R. *Methods in Ecology and Evolution*, 3(6):1084–1092, 2012.

[8] Jeremy M. Beaulieu and Brian C. O'Meara. Detecting hidden diversification shifts in models of trait-dependent speciation and extinction. *Systematic Biology*, 65(4):583–601, 2016.

[9] Daniel S. Caetano, Brian C. O'Meara, and Jeremy M. Beaulieu. Hidden state models improve state-dependent diversification approaches, including biogeographical models. *Evolution*, 72(11):2308–2324, 2018.

[10] Leonel Herrera-Alsina, Paul van Els, and Rampal S. Etienne. Detecting the dependence of diversification on multiple traits from phylogenetic trees and trait data. *Systematic Biology*, 68(2):317–328, 2018.

[11] E.A. Thompson. *Human evolutionary trees.* Cambridge University Press Cambridge, 1975.

[12] Sean Nee, Robert M. May, and Paul H. Harvey. The Reconstructed Evolutionary Process. *Philosophical Transactions: Biological Sciences*, 344(1309):305–311, 1994.

[13] Mark S. Springer, Robert W. Meredith, John Gatesy, Christopher A. Emerling, Jong Park, Daniel L. Rabosky, Tanja Stadler, Cynthia Steiner, Oliver A. Ryder, Jan E. Janečka, et al. Macroevolutionary dynamics and historical biogeography of primate diversification inferred from a species supermatrix. *PLoS One*, 7(11):e49521, 2012.

[14] Brian R Moore, Sebastian Höhna, Michael R May, Bruce Rannala, and John P Huelsenbeck. Critically evaluating the theory and performance of Bayesian analysis of macroevolutionary mixtures. *Proceedings of the National Academy of Sciences*, 113(34):9569–9574, 2016.

[15] S Höhna, MJ Landis, and TA Heath. Phylogenetic inference using RevBayes. *Current Protocols in Bioinformatics*, 57:6–16, 2017.

[16] Marjorie G. Weber and Anurag A. Agrawal. Defense mutualisms enhance plant diversification. *Proceedings of the National Academy of Sciences*, 111(46):16442–16447, 2014.

[17] Mette E. Steeman, Martin B. Hebsgaard, R. Ewan Fordyce, Simon Y. W. Ho, Daniel L. Rabosky, Rasmus Nielsen, Carsten Rahbek, Henrik Glenner, Martin V. Sørensen, and Eske Willerslev. Radiation of extant cetaceans driven by restructuring of the oceans. *Systematic Biology*, 58(6):573–585, 2009.

[18] Elizabeth L. Spriggs, Wendy L. Clement, Patrick W. Sweeney, Santiago Madriñán, Erika J. Edwards, and Michael J. Donoghue. Temperate radiations and dying embers of a tropical past: the diversification of Viburnum. *New Phytologist*, 207(2):340–354, 2015.

[19] Yanis Bouchenak-Khelladi, Renske E. Onstein, Yaowu Xing, Orlando Schwery, and H. Peter Linder. On the complexity of triggering evolutionary radiations. *New Phytologist*, 207(2):313–326, 2015.

[20] Andrew B. Leslie, Jeremy M. Beaulieu, Hardeep S. Rai, Peter R. Crane, Michael J. Donoghue, and Sarah Mathews. Hemisphere-scale differences in conifer evolutionary dynamics. *Proceedings of the National Academy of Sciences*, 109(40):16217–16221, 2012.

[21] Samantha R. Cook, Andrew Gelman, and Donald B. Rubin. Validation of software for Bayesian models using posterior quantiles. *Journal of Computational and Graphical Statistics*, 15(3):675–692, 2006.

[22] Sean Talts, Michael Betancourt, Daniel Simpson, Aki Vehtari, and Andrew Gelman. Validating Bayesian inference algorithms with simulation-based calibration. *arXiv preprint arXiv:1804.06788*, 2018.

[23] Fábio K. Mendes, Remco Bouckaert, Luiz M. Carvalho, and Alexei J. Drummond. How to validate a Bayesian evolutionary model. *Systematic Biology*, 74(1):158–175, 2025.

[24] John P. Huelsenbeck and Bruce Rannala. Frequentist Properties of Bayesian Posterior Probabilities of Phylogenetic Trees Under Simple and Complex Substitution Models. *Systematic Biology*, 53(6):904–913, 2004. doi: 10.1080/10635150490522629.

[25] Stilianos Louca and Matthew W. Pennell. Extant timetrees are consistent with a myriad of diversification histories. *Nature*, 580(7804):502–505, 2020.

[26] Sebastian Höhna. Likelihood Inference of Non-Constant Diversification Rates with Incomplete Taxon Sampling. *PLoS One*, 9(1):e84184, 2014.

[27] R.E. Kass and A.E. Raftery. Bayes factors. *Journal of the American Statistical Association*, 90:773–795, 1995.

[28] W. Xie, P. O. Lewis, Y. Fan, L. Kuo, and M-H. Chen. Improving marginal likelihood estimation for Bayesian phylogenetic model selection. *Systematic Biology*, 60:150–160, 2011.

[29] Sebastian Höhna, Michael J. Landis, and John P. Huelsenbeck. Parallel power posterior analyses for fast computation of marginal likelihoods in phylogenetics. *PeerJ*, 9:e12438, 2021.

[30] H. Jeffreys. *Theory of Probability.* Oxford University Press, Oxford University Press, 1939.

[31] M. D. Lee and E. J. Wagenmakers. *Bayesian cognitive modeling: A practical course.* Cambridge University Press, 2014.
